# A Gammaherpesvirus Uses Alternative Splicing to Regulate Its Tropism and Its Sensitivity to Neutralization

**DOI:** 10.1371/journal.ppat.1003753

**Published:** 2013-10-31

**Authors:** Bénédicte Machiels, Philip G. Stevenson, Alain Vanderplasschen, Laurent Gillet

**Affiliations:** 1 Immunology-Vaccinology Laboratory, Department of Infectious and Parasitic Diseases, Faculty of Veterinary Medicine, University of Liège, Liège, Belgium; 2 Division of Virology, Department of Pathology, University of Cambridge, Addenbrookes Hospital, Cambridge, United Kingdom; Louisiana State University, United States of America

## Abstract

Human gammaherpesviruses are associated with the development of lymphomas and epithelial malignancies. The heterogeneity of these tumors reflects the ability of these viruses to route infection to different cell types at various stages of their lifecycle. While the Epstein Barr virus uses gp42 – human leukocyte antigen class II interaction as a switch of cell tropism, the molecular mechanism that orientates tropism of rhadinoviruses is still poorly defined. Here, we used bovine herpesvirus 4 (BoHV-4) to further elucidate how rhadinoviruses regulate their infectivity. In the absence of any gp42 homolog, BoHV-4 exploits the alternative splicing of its Bo10 gene to produce distinct viral populations that behave differently based on the originating cell. While epithelial cells produce virions with high levels of the accessory envelope protein gp180, encoded by a Bo10 spliced product, myeloid cells express reduced levels of gp180. As a consequence, virions grown in epithelial cells are hardly infectious for CD14+ circulating cells, but are relatively resistant to antibody neutralization due to the shielding property of gp180 for vulnerable entry epitopes. In contrast, myeloid virions readily infect CD14+ circulating cells but are easily neutralized. This molecular switch could therefore allow BoHV-4 to promote either, on the one hand, its dissemination into the organism, or, on the other hand, its transmission between hosts.

## Author Summary

Gammaherpesviruses are highly prevalent human and animal pathogens. These viruses display sophisticated entry mechanisms, allowing them to infect different cell types inside a host but also to transmit between hosts in the presence of neutralizing antibodies. Here, we used bovine herpesvirus 4 (BoHV-4) to decipher how some gammaherpesviruses manage to do this. We found that, as function of the originating cell types, BoHV-4 is able to modify its tropism as well as its sensitivity to antibody neutralization just by controlling the alternative splicing of one of its genes. This virus therefore exploits post-transcriptional events to generate viral populations with distinct phenotypes.

## Introduction

Gammaherpesviruses are ubiquitous pathogens in human and animal populations all over the world. The best studied gammaherpesviruses, the Epstein-Barr virus (EBV) and the Kaposi's sarcoma-associated herpesvirus (KSHV), infect respectively some 90% [1] and 30% [2] of human populations. Primary infections by these viruses are usually subclinical, however, long-term carriage of these viruses can be associated with the development of various malignancies [3], [4] such as Burkitt lymphoma, nasopharyngal carcinoma, primary effusion lymphoma or Kaposi's sarcoma. The variety of these pathologies reflects the different tropisms of these viruses for distinct cell types. Understanding how these viruses orient their tropism is therefore essential for the development of efficient antiviral strategies and approaches to control the consequences of these infections.

Attachment to and penetration into the host cells are two distinct events in herpesvirus entry [5], [6]. As enveloped viruses, gammaherpesviruses enter cells by fusion with a cell membrane. While the precise mechanism of action is still unclear, the core fusion machinery is closely conserved and made of gB, gH and gL [6], although gL can be non-essential [7]. In contrast, the glycoproteins that mediate attachment and trigger fusion differ between viral species and also differ for the same virus depending on its target cell. This is well described for EBV, which for the most part infects epithelial cells and B lymphocytes [8]. Gp350 is the most abundant protein of the EBV envelope and is responsible for the attachment of the virus with high affinity to B cells [9]. On the opposite, gp350 deleted viruses are more infectious for epithelial cells [10], and gp350-specific antibodies enhance epithelial cell infection [11]. In addition to gp350, gp42 can function as a switch for EBV tropism. Gp42 binds to the human leukocyte antigen (HLA) class II [12], and determination of gp42 structures in its bound [13] and unbound [14] forms has made possible the development of a model for understanding how gp42 functions as a fusion activator. However, EBV makes both three-part gH/gL/gp42 complexes and two-part gH/gL complexes [15]. While fusion with a B cell is triggered by an interaction between gp42 and HLA class II [16], [17], entry into epithelial cells requires complexes without gp42 [15]. Interestingly, HLA class II expression in the virus-producing cells alters the ratio of three-part to two-part complexes. Therefore, virus produced in epithelial cells is more infectious for B cells whereas B-cell-derived virus is more infectious for epithelial cells [18]. Presumably, this switch in tropism favors the movement between epithelial cells and B cells during the cycle of persistence [19].

The mechanisms that regulate cell tropism are less clear for KSHV and other rhadinoviruses such as murid herpesvirus 4 (MuHV-4) or bovine herpesvirus 4 (BoHV-4). However, while these viruses do not have any gp42 homolog, they all encode a virion glycoprotein positionally homologous to EBV gp350/220 (BLLF1 gene). These proteins are K8.1A encoded by K8.1 in KSHV [20], gp150 encoded by MuHV-4 M7 [21] and gp180 encoded by BoHV-4 Bo10 [22]. Similarly to gp350, these proteins are involved in binding to some receptors on target cells. Indeed, K8.1A, gp150 and gp180 interact with glycosaminoglycans (GAGs) [22]–[24]. Moreover, these proteins seem to block the infection of cells that do not express this receptor [22], [23]. It has therefore been proposed that these proteins might regulate viral tropism both positively and negatively depending on the presence or the absence of their receptor [22], [23], [25]. While this putative model explains GAG+ cells infection, it does not encompass explanation of the infection of GAG-, cells such as B cells or monocytes, which are the cells that participate in the dissemination of these viruses *in vivo* [26], [27]. Therefore the question of how rhadinoviruses infect these cells remains controversial.

In this study, we showed that the BoHV-4 Bo10 gene encodes two different mRNAs through alternative splicing and that the cell-type regulated expression of one or the other transcript leads to production of virions that are phenotypically distinct. Thus, similarly to EBV, BoHV-4 progenies derived from different cell types differ in their cell tropism. Moreover, they also differ in their susceptibility to neutralizing antibodies. While epithelial virions are hardly infectious for circulating cells, they are relatively resistant to neutralization. In contrast, myeloid virions readily infect CD14+ circulating cells but are easily neutralized. We therefore propose that by regulating Bo10 mRNA splicing, BoHV-4 could promote either, on the one hand, its dissemination into the organism, or, on the other hand, its transmission between hosts.

## Results

### Bo10 mRNA undergoes alternative splicing

**Figure 1:**
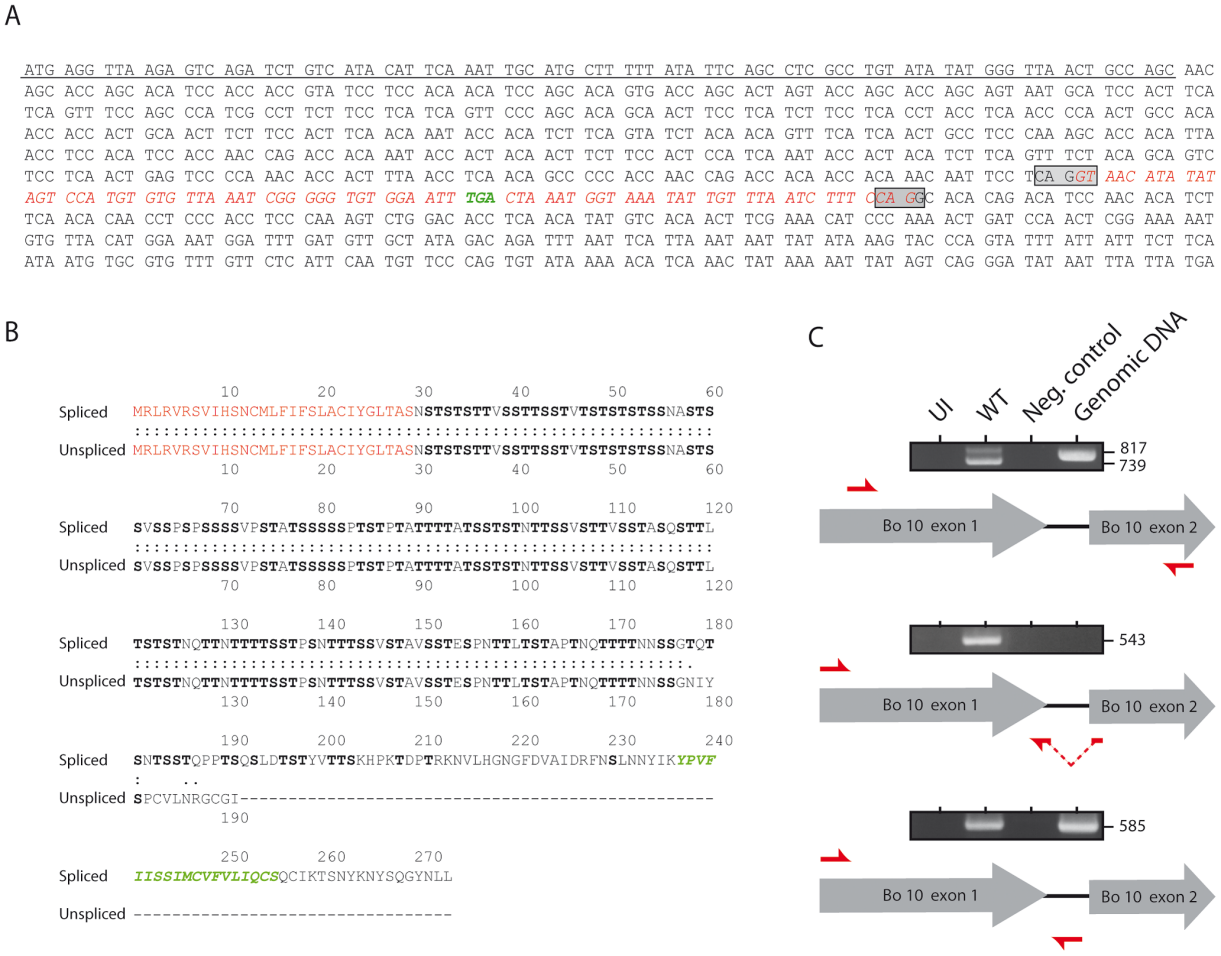
Bo10 mRNA undergoes alternative splicing. **A.** Nucleotide sequence of BoHV-4 Bo10. The underlined nucleotides represent the sequence encoding the predicted signal peptide. The boxes indicate the splicing donor and acceptor sites. The sequence in red italics indicates the nucleotides removed after splicing. Nucleotides in green indicate the STOP codon in the intron. **B.** Amino acid comparisons of the predicted spliced and unspliced Bo10 products. The red amino acids represent the signal peptide sequence. The green amino acids indicate the predicted transmembrane region of the Bo10 spliced expression product. Serines and threonines are highlighted in bold. **C.** RT-PCR analysis of the BoHV-4 Bo10 reading frame. MDBK cells were mock infected or infected with the BoHV-4 V.test strain at a MOI of 1. Twenty-four hours p.i., expression of Bo10 was studied using different pairs of primers specific for the spliced and/or the unspliced Bo10 products. Red arrows indicate locations of the different primers. Uninfected cell samples (UI), reactions without reverse transcriptase (Neg. control) and amplification of viral genomic DNA are provided as controls. Sizes in bp are indicated on the right.

Initial DNA sequence analysis of the BoHV-4 genome identified an ORF between ORF50 and ORF52 that was made of two exons [28]. With respect to its relative position in the genome, Bo10 is therefore similar to K8.1 of KSHV [20] and BLLF1 of EBV [29]. These two genes encode transmembrane glycoproteins that have been shown to be translated from a spliced message. Recently, we demonstrated that a Bo10 mRNA splicing removes 77 nucleotides and generates an mRNA encoding a 273 aa protein with a signal sequence and a membrane anchor [22]. Interestingly, sequence analysis revealed the presence of an in-frame STOP codon inside the Bo10 intron (Figure 1A) that could generate a protein (Figure 1B) similar to the potential expression product of the K8.1γ message [20]. To verify the transcription of this unspliced Bo10 message during the BoHV-4 cycle, we used RT-PCR with different pairs of primers (Figure 1C) on cDNA from BoHV-4 infected Madin-Darby bovine kidney (MDBK) cells. Primers spanning the intron revealed the existence of two different Bo10 messages. As previously described, the major 739 bp PCR product (Figure 1C) corresponded to the expected size of the product generated from the spliced mRNA. However, although weaker, an unspliced PCR product (817 bp) was also detectable (Figure 1C). RT-PCR with primer pairs specific to either the spliced or the unspliced sequence confirmed the existence of both Bo10 messenger RNAs (Figure 1C). None of the fragments could be amplified without prior reverse transcription (Neg. control lines) and DNA sequencing confirmed the identity of the different PCR products (data not shown). Altogether, these results demonstrate that the BoHV-4 Bo10 gene undergoes alternative splicing.

### Generation of the Bo10 MuDir and spliced BoHV-4 mutants

**Figure 2:**
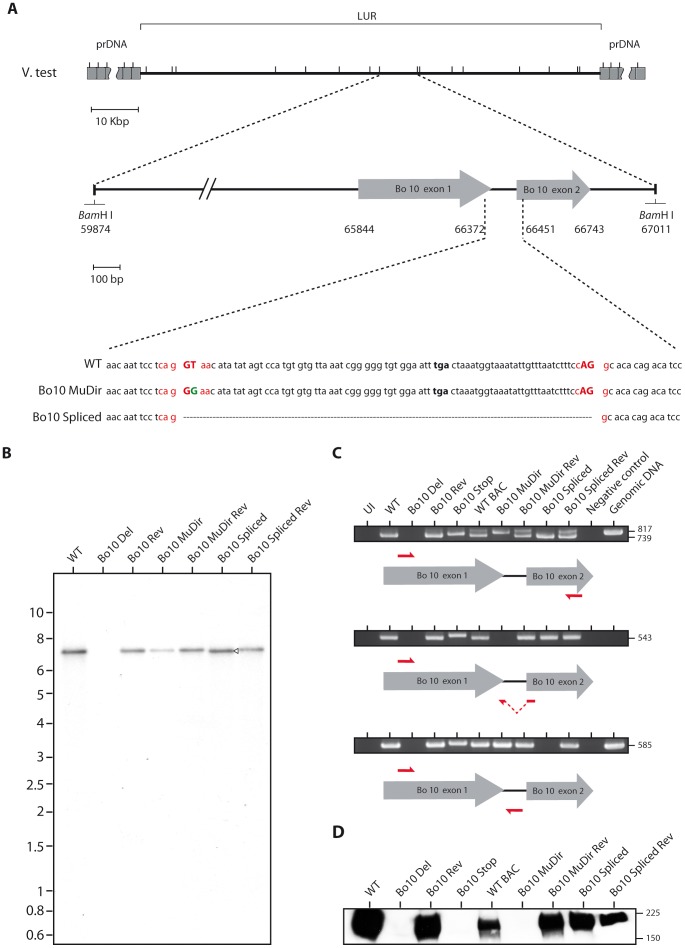
Generation of the Bo10 MuDir and Spliced BoHV-4 mutant. **A.** Schematic representation of the strategy followed to produce the recombinant BoHV-4 strains. We modified the BoHV-4 V.test Bo10 coding sequence (genomic coordinates 65844 to 66743) either by introducing a point mutation in the splicing donor site (Bo10 MuDir strain) or by replacing the entire Bo10 ORF by a sequence devoid of the intron (Bo10 Spliced strain). Splicing donor and acceptor sites are in red with splicing essential nucleotides in upper cases. The mutated nucleotide is in green. **B.** Verification of the molecular structure. Viral DNA was digested with *Bam*HI, resolved by agarose gel electrophoresis, and hybridized with a ^32^P-labeled probe, corresponding to nucleotides 65900–66370 of the BoHV-4 V.test strain genome (coding for Bo10 Exon 1). The 7,137-bp wild-type (WT) band becomes 7060-bp (open arrow) for the Bo10 spliced mutant. Marker sizes in Kbp are indicated on the left. **C.** RT-PCR analysis of BoHV-4 Bo10 expression by the different mutants. MDBK cells were mock infected or infected with the different BoHV-4 mutant strains at a MOI of 1. Twenty-four hours p.i., expression of Bo10 was studied using different pairs of primers specific for the spliced and/or unspliced Bo10 cDNA. Uninfected cell samples (UI), reactions without reverse transcriptase (Neg. control) and amplification of viral genomic DNA are provided as controls. Sizes in bp are indicated on the right. **D.** Detection of the Bo10 encoded gp180 protein by the anti-Bo10-c15 serum. Purified virions (5*10^5^ virions per lane) were subjected to western blotting with anti-Bo10-c15 serum as described in the Methods. The position of a molecular mass (MM) standard (in kDa) is shown on the right.

We previously described two BoHV-4 Bo10 knockout strains [22], [30]. To unravel the function of both the spliced and the unspliced Bo10 expression products, we generated two additional Bo10 mutant viruses. In the Bo10 MuDir mutant, we punctually mutated the Bo10 splicing donor site (T to G) to only express the unspliced form. In contrast, the Bo10 Spliced strain only expresses the Bo10 spliced product (Figure 2A). Southern blots of viral DNA (Figure 2B) confirmed the expected genomic structures of the two mutants and their associated revertant strains. The expected mutations were further confirmed by DNA sequencing (data not shown). As expected, RT-PCR analysis showed that the Bo10 MuDir and the Bo10 Spliced strains express the unspliced or the spliced messenger RNA respectively (Figure 2C). Finally, immunoblotting with an anti-Bo10-c15 rabbit polyserum confirmed that only the Bo10 MuDir mutant virions lacked gp180, encoded by the spliced Bo10 product (Figure 2D) while content of other proteins such as gB appeared to be normal (Figure S1).

### Effect of Bo10 mRNA splicing on BoHV-4 replication *in vitro*

**Figure 3:**
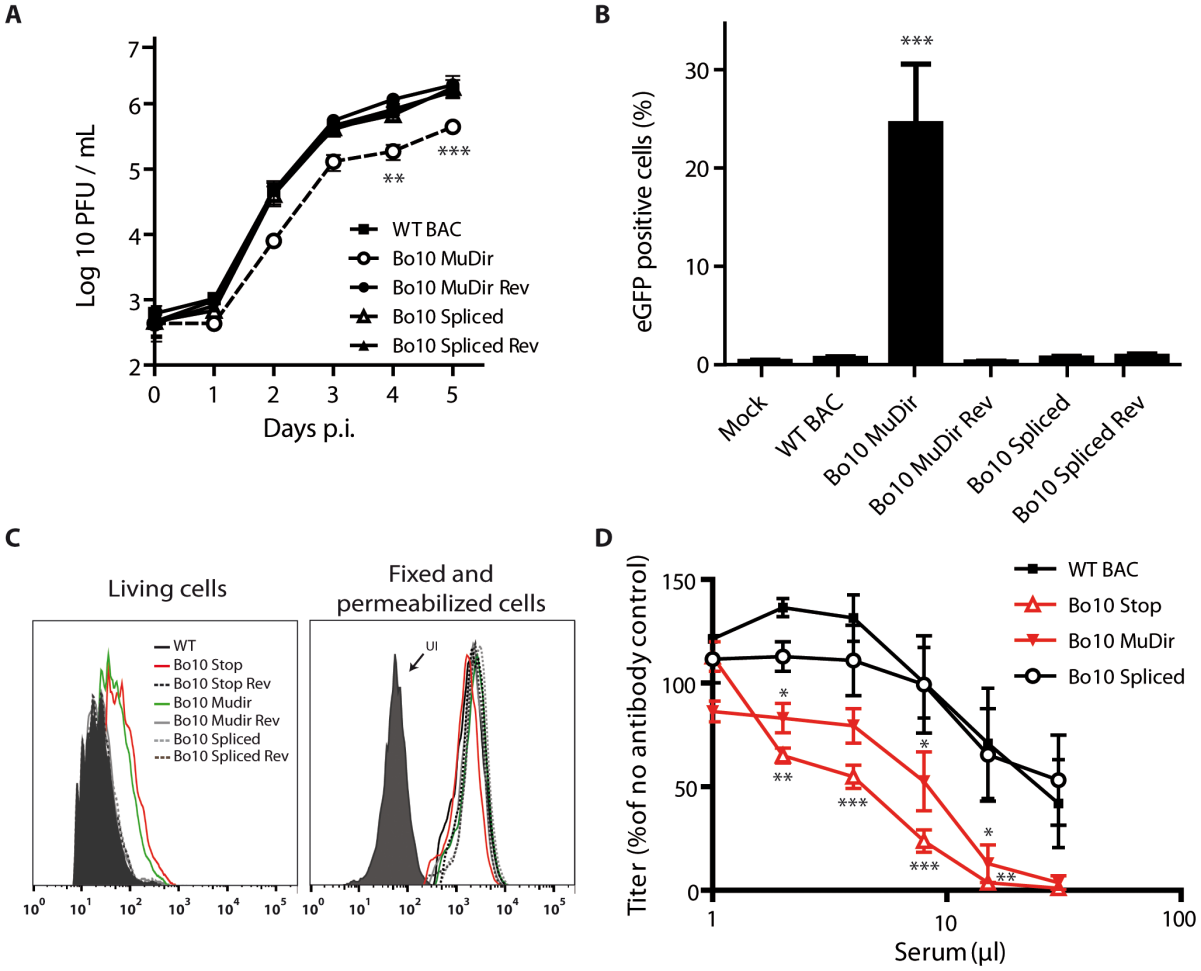
Bo10 mRNA splicing determines phenotype of BoHV-4 virions. Effect of Bo10 mRNA splicing on BoHV-4 tropism (A–B) and antibody recognition (C–D). **A.** MDBK cells grown in 6-well cluster dishes were infected at a MOI of 0.01 for multi-step assays as described in the Methods with BoHV-4 WT BAC (black squares), Bo10 MuDir (open circles), Bo10 MuDir Rev (black circles), Bo10 Spliced (open triangles) and Bo10 Spliced Rev (black triangles). Supernatant of infected cultures and infected cells were harvested at different times p.i. and the amount of infectious virus was determined by plaque assay on MDBK cells. Time 0 p.i. is re-titration of the inocula to ensure that similar amounts of virus were put on the cells. Plaques were visualized by immunofluorescent staining as described in the Methods. The data presented are the average ± SEMs for triplicate measurements. The data were analyzed by 2way ANOVA and Bonferroni posttests, ** p<0.01, *** p<0.001. **B.** Cattle PBMCs were infected with WT BAC, Bo10 MuDir, Bo10 MuDir Rev, Bo10 Spliced and Bo10 Spliced Rev strains (1 PFU/cell). Twenty-four hours later, cells were analyzed by flow cytometry for CD14 and viral eGFP expression as described in the Methods. The data presented are the average ± SEMs for 6 measurements and were analyzed by 1way ANOVA and Bonferroni posttests, *** p<0.001. **C.** MDBK cells were infected (2 PFU/cell, 36 h) with WT (solid black lines), Bo10 STOP (red lines), Bo10 Stop Rev (dotted black lines), Bo10 MuDir (green lines), Bo10 MuDir Rev (grey lines), Bo10 Spliced (dotted grey lines) or Bo10 Spliced Rev (dotted dark grey lines) of BoHV-4 V.test, and then analyzed by flow cytometry for gB detection (mAb 29). The filled histogram shows uninfected cells (UI). Fixed (PFA 4%, 4°C for 30 min) and permeabilized (saponin 0.1%) cells were used as controls of protein expression. **D.** BoHV-4 WT BAC, Bo10 STOP, Bo10 MuDir and Bo10 Spliced virions were incubated with sera of 3 different rabbits infected with BoHV-4 V.test strain. After incubation (2 h, 37°C), the viruses were plaque assayed for infectivity on MDBK cells. BoHV-4 titers are expressed relative to virus without antibody. The data presented are the average ± SEMs for 3 measurements and were analyzed by 2way ANOVA and Bonferroni posttests, * p<0.05, ** p<0.01, *** p<0.001.

We previously showed that the Bo10 knockout strains display a growth deficit associated with reduced binding to epithelial cells. We interpreted this phenotype as being a consequence of the absence of the gp180 glycoprotein that is encoded by the Bo10 spliced message. However, as the Bo10 gene encodes two different transcripts, this growth deficit could also be associated with the absence of the unspliced Bo10 message. To address this question, multi-step growth assays were performed on MDBK cells with the different Bo10 mutants. Interestingly, only the Bo10 MuDir virus grew to lower titers than the WT BAC parental strain (Figure 3A).

### Enhanced infection of GAG− cells by the BoHV-4 Bo10 MuDir strain

With the Bo10 knockout strains, we interpreted the growth deficit on epithelial cells as a consequence of reduced binding to GAG [22]. Moreover, we showed that Bo10 deletion enhanced infection of GAG-deficient cells [22]. We therefore compared the relative dependence on GAGs of the different BoHV-4 strains constructed in this study by infecting CHO-K1 fibroblasts competent or not for GAG expression (Figure S2). As observed with the Bo10 deficient-viruses, Bo10 MuDir virions infected CHO GAG+ cells similarly to the WT virus but infected CHO GAG− cells much better (Figure S2). The Bo10 spliced virions (expressing gp180) behaved similarly to WT virions (Figure S2). *In vivo*, BoHV-4 infects monocytes, which are, however, relatively GAG deficient [31]. We therefore compared the capacity of the different viruses to infect rabbit (Figure S3) and cow (Figure 3B) peripheral blood mononuclear cells (PBMCs) *ex vivo*. While all the other strains infected rabbit and cow CD14+ PBMCs very poorly, Bo10 MuDir viruses infected them much better (Figure S3 and 3B).

### Bo10 splicing regulates BoHV-4 antigenicity and susceptibility to neutralization

We have previously shown that gp180 provides part of a glycan shield for otherwise vulnerable viral epitopes [30]. Indeed, antibodies have greater access to gB, gH and gL on Bo10 knockout virions and this correlates with a greater susceptibility to neutralization by immune sera [30]. As we have shown that Bo10 encodes two transcripts, we used Bo10 spliced and Bo10 MuDir mutants to investigate which of these transcripts is responsible for antibody evasion. Infected cell surfaces provide a means of probing antigenic differences between BoHV-4 glycoprotein mutants. We therefore compared epitope accessibility on cells infected by the different BoHV-4 Bo10 mutant viruses. Only the cells infected by the Bo10 MuDir and Stop mutants were more heavily stained with mAb 29 recognizing gB (Figure 3C). This result was not due to differences in protein expression, as permeabilized cells gave similar staining with each virus (Figure 3C). It therefore appears that only the spliced Bo10 transcript, encoding gp180, is involved in shielding other epitopes at the cell surface.

We therefore further compared the sensitivity of BoHV-4 WT BAC, Bo10 STOP, Bo10 MuDir and Bo10 Spliced strains to neutralization by sera of rabbits infected with the BoHV-4 V.test strain (Figure 3D). WT and Bo10 Spliced virions were poorly neutralized. In contrast, Bo10 STOP and Bo10 MuDir virions were much more efficiently neutralized. Especially, complete neutralization was now possible. Thus, Bo10 splicing and the subsequent gp180 expression are critical to limiting virion neutralization.

### Bovine epithelial and myeloid cells produce different kinds of BoHV-4 virions

**Figure 4:**
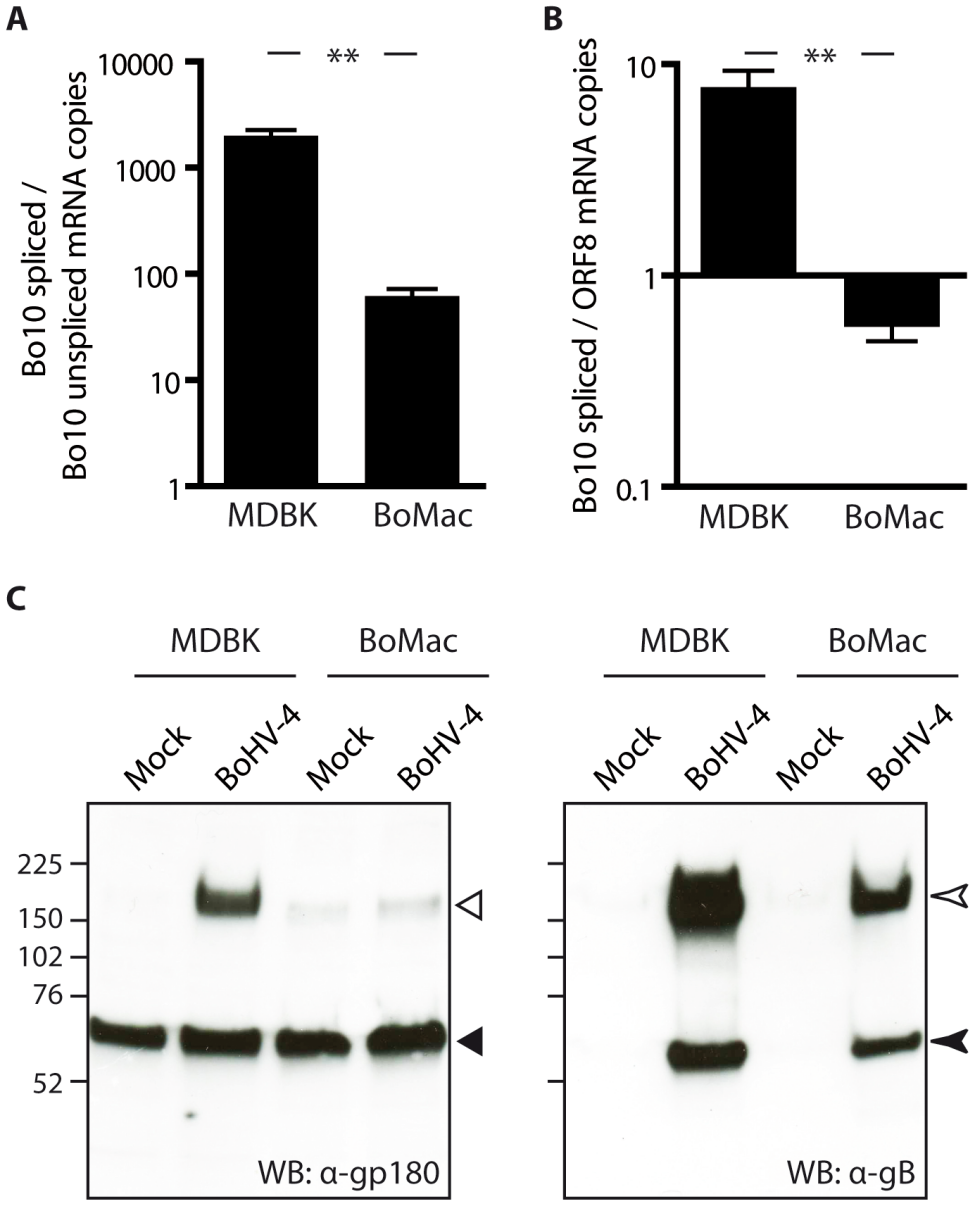
BoMac myeloid cells express relatively less gp180 glycoprotein in comparison with epithelial cells. **A–B**. MDBK and BoMac cells were infected with the BoHV-4 V.test strain at a MOI of 1. Twenty-four hours p.i., relative expressions of Bo10 spliced *vs* unspliced transcripts (**A**) and of Bo10 spliced *vs* ORF8 transcripts (**B**) were estimated as described in the Methods. The data presented are the average ± SEMs for 3 measurements and were analyzed by Student's t-test, ** p<0.01. **C.** Detection of the gp180, encoded by the Bo10 spliced transcript, and gB glycoproteins in bovine epithelial and myeloid cells. MDBK and BoMac cells were infected with the BoHV-4 V.test strain at a MOI of 1. Twenty-four hours p.i., these cells were subjected to western blotting with anti-Bo10-c15 serum (recognizing gp180) and mAb 35 (recognizing gB) as described in the Methods. On the gp180 blot, open and filled triangles indicate respectively the specific 180 kDa protein and a background band. On the gB blot, open and filled arrow heads indicate uncleaved gB and furin-cleaved gB C-terminus respectively. The position of a molecular mass (MM) standard (in kDa) is shown on the left.

Different bovine cell lines were then investigated for expression of Bo10 spliced and unspliced transcripts by quantitative PCR. In order to be able to compare the numbers of both transcripts, a plasmid encoding both Bo10 spliced and unspliced specific sequences was constructed and used as a unique standard curve for both reactions. Interestingly, while the infected epithelial MDBK cells displayed around 1000 times more Bo10 spliced transcript than unspliced transcript, this ratio was more than 10 times lower in the BoMac myeloid cells (Figure 4A). We then compared the number of Bo10 spliced transcripts, encoding gp180, to the number of ORF8 transcripts, encoding gB, as we have shown that one of the glycoproteins shielded by gp180 is gB (Figure 3C) [30]. While the infected epithelial MDBK cells displayed around 10 times more Bo10 spliced transcripts than ORF8 transcripts, this ratio was around 10 times lower in the BoMac myeloid cells (Figure 4B), suggesting that in these cells, there could be less expression of gp180 glycoprotein in comparison to gB. Similar results were obtained when the amount of Bo10 spliced transcripts was compared to the number of ORF47 mRNA encoding gL (Figure S4), as gL is another viral glycoprotein shielded by gp180. Finally, these results were confirmed by western blotting on MDBK or BoMac cells (Figure 4C). Interestingly, while gB glycoprotein was readily detectable in both cell types, almost no gp180 glycoprotein was observed in BoHV-4 WT infected BoMac cells.

**Figure 5:**
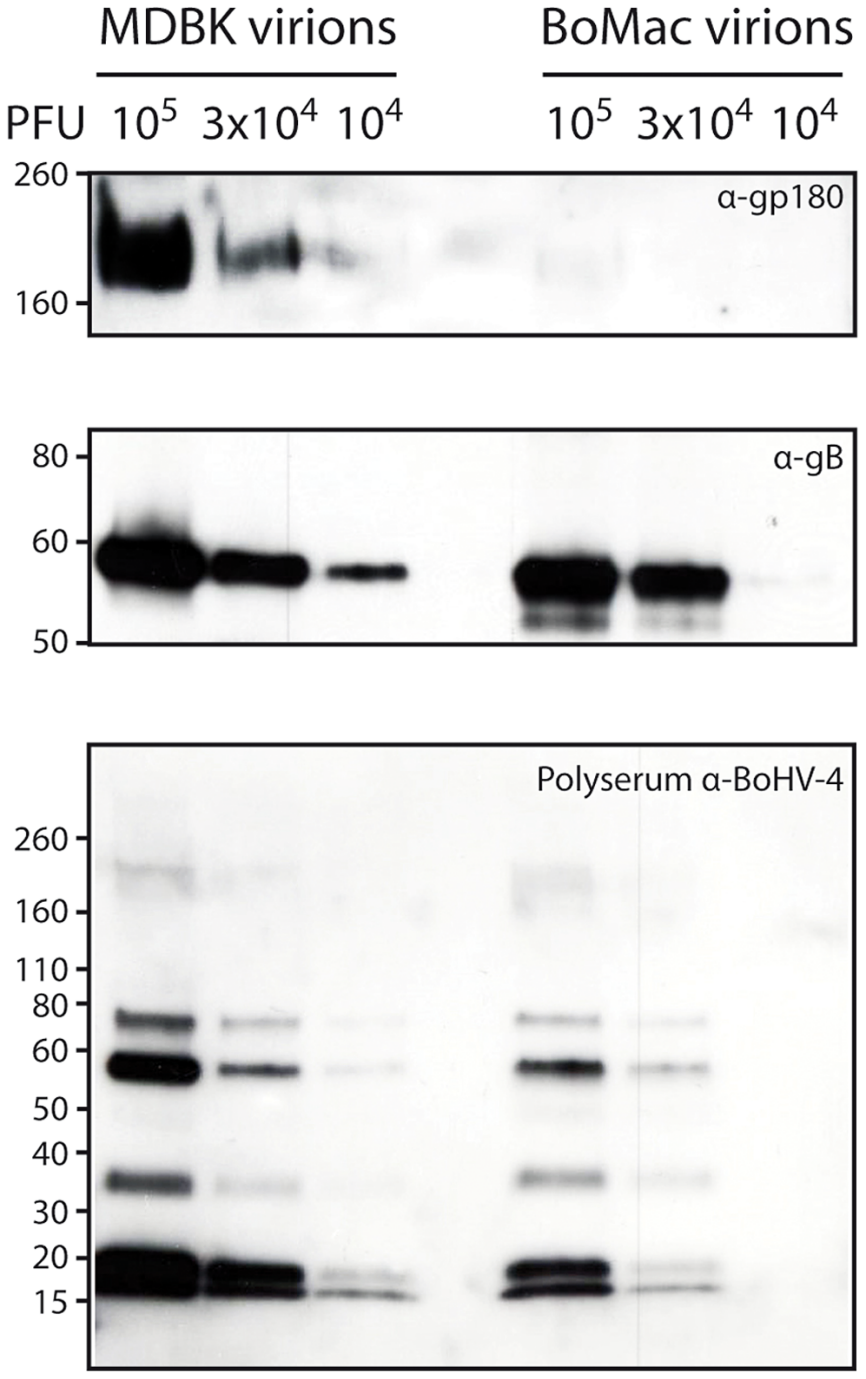
Bovine epithelial and myeloid cells produce different kinds of BoHV-4 virions. Detection of the gp180 and gB glycoproteins in MDBK and BoMac virions. Different amounts of MDBK and BoMac virions were compared for gp180 (with anti-Bo10-c15 serum) and gB (with mAb 35) content per PFU by immunoblotting as described in the Methods. Anti-BoHV-4 polyserum was taken as control. For each blot, the position of a molecular mass (MM) standard (in kDa) is shown on the left.

Finally, incorporation of gp180 in MDBK and BoMac virions was assessed by immunoblotting on purified virions. As observed on infected cells, BoMac virions contain relatively less gp180 than MDBK virions while amounts of gB appear to be similar (Figure 5). Blotting with a rabbit polyserum raised against BoHV-4 virions was used as loading control. These results therefore suggest that the BoHV-4 phenotype could change with the type of cell in which it is grown, namely epithelial or myeloid cells.

### Phenotypic changes in myeloid cell-derived virions

**Figure 6:**
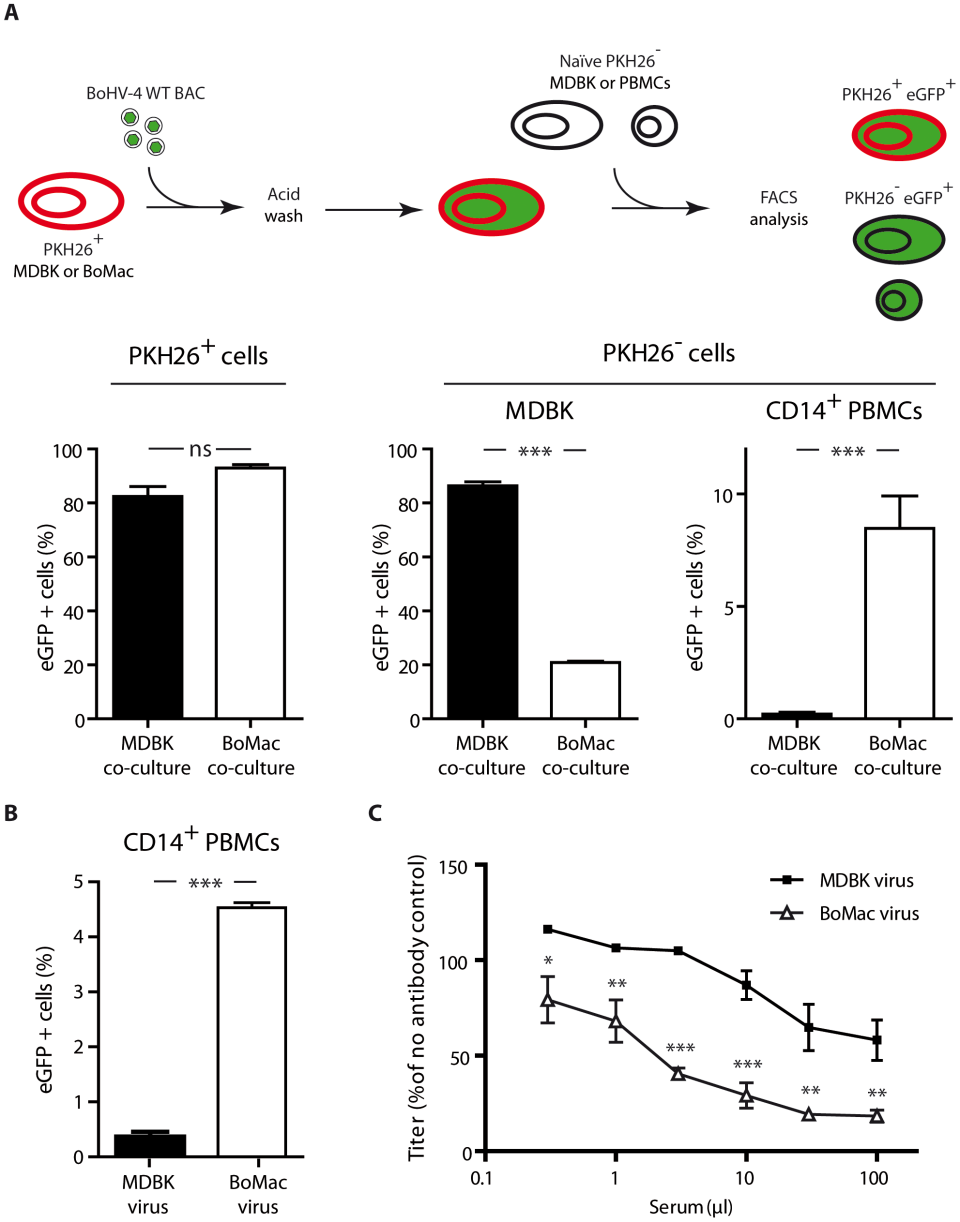
Myeloid virions are more infectious for CD14+ PBMCs but are more sensitive to antibody neutralization. **A.** Infection by co-cultures. MDBK or BoMac cells previously stained with the membrane dye PKH26 were infected with BoHV-4 WT BAC virions (MOI of 1). Three hours p.i., cells were washed with acidic solution (PBS pH 3) in order to inactivate and remove the inoculum, and 12 hours p.i., freshly isolated PBMCs or MDBK controls were added. After 24 hours of co-cultivation, cells were collected and the proportion of eGFP expressing cells was measured in PKH26+ and PKH26− cells. For PBMC co-cultures, the proportion of eGFP expressing cells was measured in PKH26− CD14+ cells. The data presented are the average ± SEMs for 5 measurements and were analyzed by Student's t-test, *** p<0.001. **B.** BoHV-4 WT BAC cell-free virions propagated on MDBK or BoMac cells were added to bovine PBMCs at a MOI of 1 according to titers measured on MDBK cells. 24 hours later, percentages of eGFP positive cells were measured in CD14+ PBMCs by flow cytometry. The data presented are the average ± SEMs for 5 measurements and were analyzed by Student's t-test, *** p<0.001. **C.** BoHV-4 WT BAC virions propagated on MDBK or BoMac cells were incubated with sera of 3 different rabbits infected with BoHV-4 V.test strain (propagated on MDBK cells). After incubation (2 h, 37°C), the viruses were plaque assayed for infectivity on MDBK cells. BoHV-4 titers are expressed relative to virus without antibody. The data presented are the average ± SEMs for 3 measurements and were analyzed by 2way ANOVA and Bonferroni posttests, * p<0.05, ** p<0.01, *** p<0.001.

We first compared phenotypes of MDBK and BoMac derived virions by co-culture experiments (Figure 6A). While naïve MDBK cells co-cultured overnight with BoHV-4 WT BAC infected MDBK cells became >80% eGFP^+^, CD14+ PBMCs cultured under the same conditions remained nearly entirely uninfected (<1% eGFP^+^) (Figure 6A). In contrast, BoHV-4 WT BAC infected BoMac cells produced lower amount of virions, as demonstrated by the fact that only ∼20% of co-cultured MDBK cells became eGFP^+^. However, these few virions were much more infectious for CD14+ PBMCs (>10% eGFP^+^) in comparison with MDBK derived virions (Figure 6A). Therefore direct contact with infected myeloid BoMac cells allowed efficient BoHV-4 infection of circulating CD14+ PBMCs.

We next tested whether cell-free virions derived from myeloid BoMac cells could also better infect CD14+ PBMCs compared to cell-free virions derived from epithelial MDBK cells. While MDBK-derived virions barely infected 0.5% of PBMCs at the multiplicity of infection (MOI) of 3, according to titers measured on MDBK cells, BoMac-derived virions infected these cells substantially more efficiently as around 5% of CD14+ PBMCs became positive (Figure 6B).

The second phenotypic difference between Bo10 MuDir and Spliced virions was related to resistance to neutralization by immune sera. Therefore, we compared sensitivity to serum neutralization of BoHV-4 WT BAC virions derived from MDBK or BoMac cells. As previously observed [30], MDBK-derived WT virions were poorly neutralized by immune sera. In contrast, BoMac-derived WT virions were neutralized much more efficiently (Figure 6C).

**Figure 7:**
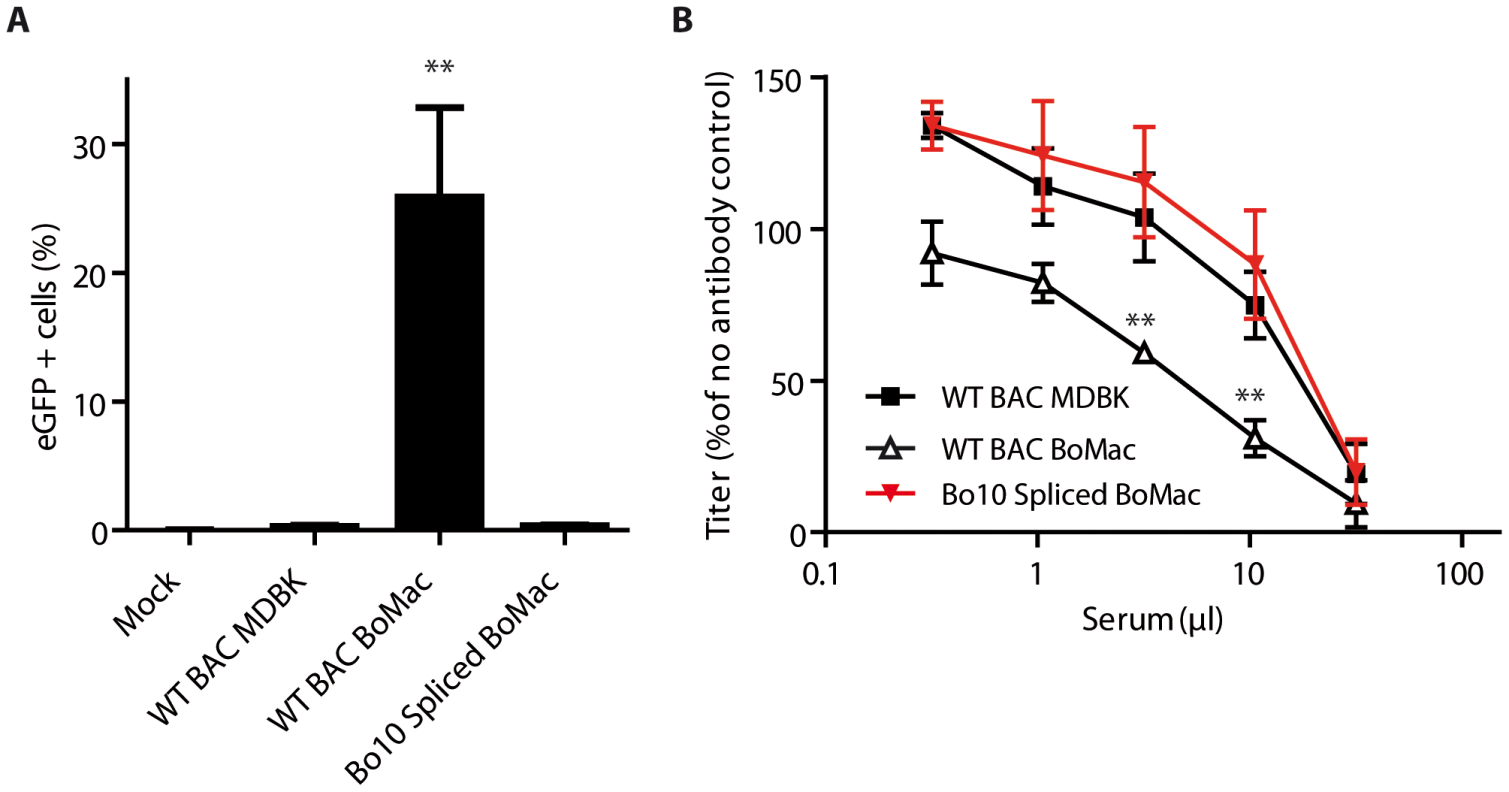
Expression of the spliced form of Bo10 mRNA reverts the phenotype of myeloid virions. **A.** BoHV-4 WT BAC or Bo10 Spliced virions were propagated on MDBK or BoMac cells and then added to bovine PBMCs at a MOI of 3 according to titers measured on MDBK cells. 24 hours later, percentages of eGFP positive cells were measured in CD14+ PBMCs by flow cytometry. The data presented are the average ± SEMs for 4 measurements and were analyzed by 1way ANOVA and Bonferroni posttests, ** p<0.01. **B.** BoHV-4 WT BAC or Bo10 Spliced virions propagated on MDBK or BoMac cells were incubated with sera of 3 different rabbits infected with BoHV-4 V.test strain (propagated on MDBK cells). After incubation (2 h, 37°C) the viruses were plaque assayed for infectivity on MDBK cells. BoHV-4 titers are expressed relative to virus without antibody. The data presented are the average ± SEMs for 3 measurements and were analyzed by 2way ANOVA and Bonferroni posttests, ** p<0.01.

Finally, in order to demonstrate that the phenotypic difference of BoHV-4 myeloid virions was mainly due to reduced Bo10 mRNA splicing, Bo10 Spliced virions were propagated on BoMac cells and compared to WT BAC virions derived from MDBK or BoMac cells (Figure 7). While WT BAC BoMac-derived virions readily infected CD14+ PBMCs, Bo10 Spliced virions derived from BoMac cells infected very few CD14+ PBMCs similarly to WT BAC virions propagated on MDBK cells (Figure 7A). Moreover, in contrast to BoMac-derived WT virions, Bo10 Spliced virions propagated on BoMac cells resisted neutralization similarly to BoHV-4 WT BAC virions derived from MDBK cells.

Altogether, these results show that regulation of Bo10 mRNA splicing offers the possibility of regulating both the tropism and the antigenicity of BoHV-4 virions.

## Discussion

Herpesvirus lifecycles are probably among the most complex lifecycles of all viruses. Indeed, these viruses are able not only to engender either latent or lytic infections but are also able to do so in different cell types at different stages of infection. This is particularly true of gammaherpesviruses, for which viral replication in mucosal epithelium appears to be mainly important for host entry [32] and exit [33], [34], whereas latency establishment in circulating leukocytes ensures host colonization [35]. Infection of these two key targets has proven to follow substantially different pathways [8] and regulation of these processes may allow these viruses to route infection *in vivo*. For example, EBV appears to use gp42 as a switch of cell tropism [18]: epithelial cells produce virions high in gH/gL/gp42 complexes, which promote B-cell infection, while B cells produce viruses low in gp42, which efficiently infect epithelial cells but not B cells.

While rhadinoviruses, such as KSHV or BoHV-4, share a similar alternate tropism *in vivo*, the mechanisms underlying this property were still unknown. Thus, BoHV-4 persists in circulating CD14+ cells *in vivo*, yet infects them poorly *in vitro*. In this study, we showed that alternative splicing of the Bo10 gene defines the cell tropism of BoHV-4 virions. In addition to the Bo10 spliced message, that encodes gp180, an unspliced Bo10 mRNA is transcribed during the BoHV-4 cycle (Figure 1). Viral strains expressing one or the other transcript showed different infectivity patterns associated with GAG expression (Figure S2). Exclusive expression of the unspliced transcript generated virions that were both less infectious for GAG+ cells than the wild-type and more infectious for GAG− cells. On the opposite, exclusive expression of the spliced message blocked GAG− cells infection. Moreover, relative proportions of these two transcripts determined the release of distinct viral populations. Epithelial cells, expressing high amounts of gp180, produced virions that were unable to infect CD14+ in contrast to virions derived from myeloid cells, expressing low amounts of gp180 (Figures 4–6).

**Figure 8:**
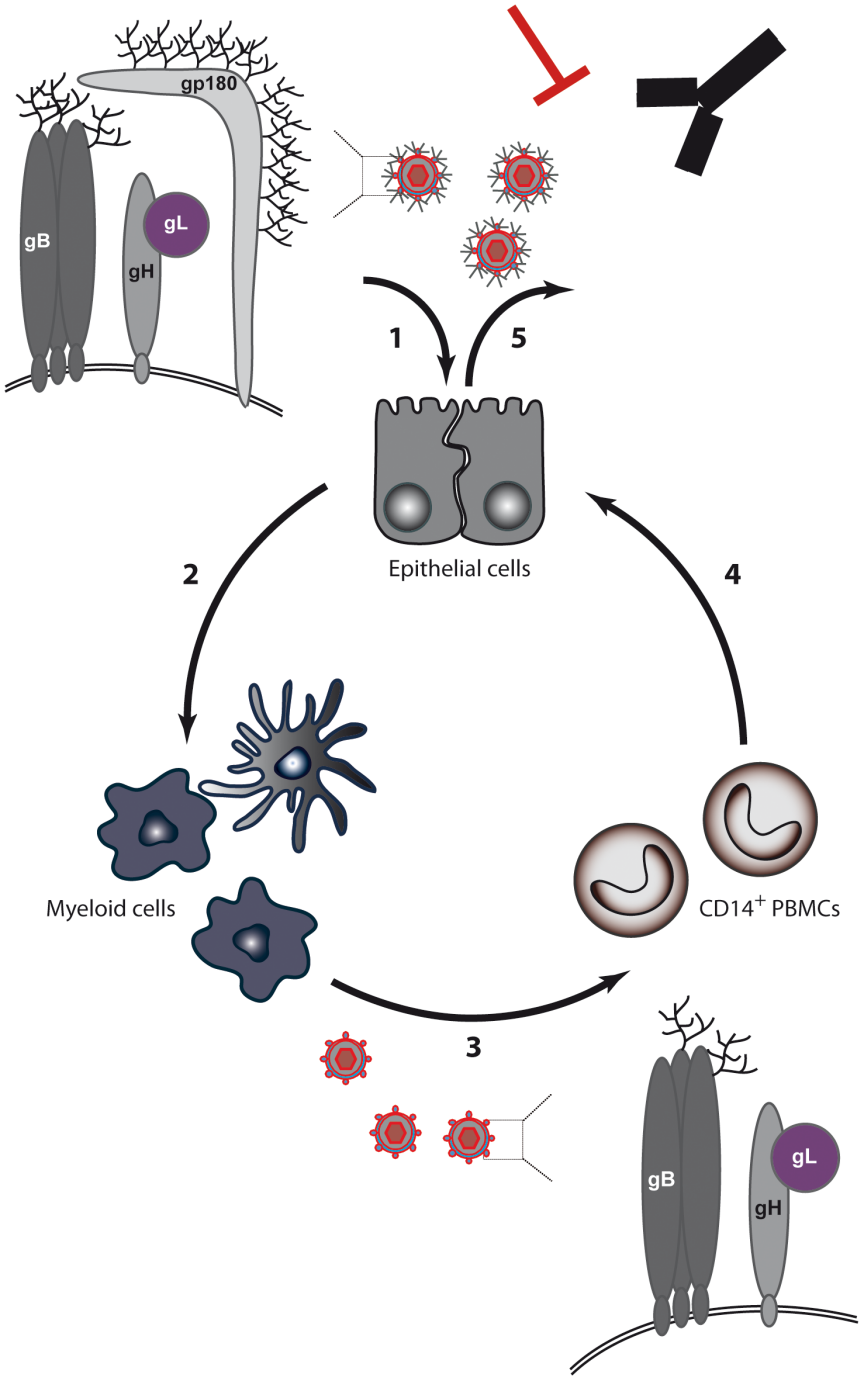
BoHV-4 model for switch of cell tropism through alternative splicing of the Bo10 gene. (1) Expression of the Bo10 spliced product, encoding gp180, positively regulates the infection of GAG-bearing cells. (2) Replication in epithelial cells likely allows infection of myeloid cells. (3) Myeloid infection allows infection of circulating leukocytes, such as CD14+ cells in the case of BoHV-4. (4) Infected PBMCs transmit infection to epithelium of the excretion sites by an unknown route. (5) Finally, incorporation of gp180 at the excretion site allows virions to be protected from neutralizing antibodies.

These results suggest an epithelial/myeloid/circulating leukocyte infection pathway for BoHV-4 (Figure 8), similar to the one recently described for MuHV-4 [36]. Indeed, we had previously showed that the BoHV-4 Bo10 gene positively regulates the infection of GAG-bearing cells such as epithelial cells [22]. In this study we showed that this is through the expression of the Bo10 spliced product, encoding gp180. *In vivo*, such interaction probably occurs at specific locations displaying accessible heparin sulfates structures such as the olfactory neuroepithelium as shown for MuHV-4 [32]. Replication in epithelial cells likely allows infection of some myeloid cells which are susceptible to infection by epithelial virions. Finally, this myeloid infection allows infection of circulating leukocytes, such as CD14+ cells in the case of BoHV-4. Interestingly, a similar role of myeloid infection has been proposed in the case of MuHV-4 [36]. However, differences exist between the two models. Firstly, MuHV-4 infects mainly circulating B cells. Secondly, RT-PCR has never demonstrated MuHV-4 gp150 truncation by splicing nor were myeloid cell-derived MuHV-4 virions gp150-deficient [36]. Moreover, the results obtained with MuHV-4 suggested that the myeloid-derived virions phenotype could be associated with conformational changes in gB and gH. While this cannot be excluded in our model, the fact that the forced expression of gp180 in myeloid cells, as seen with Bo10 Spliced virions (Figure 7), or the absence of expression of gp180 in epithelial cells, as seen with Bo10 MuDir virions (Figure 3B), is sufficient to switch cell tropism suggest that Bo10 alternative splicing is the main determinant of BoHV-4 virion tropism. While our main hypothesis is that gp180 is directly involved in switching the tropism of BoHV-4 virions (direct effect), we cannot rule out that the amount of gp180 that is incorporated in virions affects either the recruitment of another protein or its conformation (indirect effect).

When seen only from the angle of cell tropism, the role of gp180 appears unclear. Indeed, it is unnecessary for growth on epithelial cells and it negatively regulates infection of GAG− cells (Figures 3, S2 and S3). Moreover, a BoHV-4 strain deleted for the Bo10 gene displays no latency establishment deficit *in vivo* [30]. Positive selection pressure for gp180 expression is therefore difficult to understand in these contexts. As was recently demonstrated for influenza, the evasion of neutralizing antibodies is one of the main forces that drive virus evolution [37]. This is particularly true for gammaherpesviruses that establish lifelong latency and are therefore continuously shed in the presence of neutralizing antibodies [34]. In this context, we recently showed that the deletion of BoHV-4 Bo10 gene markedly sensitized virions to neutralization by immune sera. The results obtained here demonstrate that this phenomenon is related, either directly or indirectly, to gp180 expression, as only Bo10 MuDir virions and not Bo10 spliced virions displayed increased sensitivity to neutralization (Figure 3D). Similarly, WT virions derived from myeloid cells were more easily neutralized than epithelial cell-derived WT virions (Figure 6C). Again, this was associated with reduced expression of the spliced Bo10 transcript, as the forced expression of gp180 in myeloid cells substantially reduced sensitivity to neutralization by immune sera (Figure 7B). These observations make sense in the context of an *in vivo* cycle as epithelial cell-derived virions have to face neutralizing antibodies that are likely to be present at mucosal sites. In contrast, because spread of a gammaherpesvirus within the host is more likely to involve direct cell–cell contact [38] rather than cell-free virion release, myeloid cell-derived virions may not have to face a similar constraint. Epithelial virions would therefore be better fit for virus transmission between hosts, while myeloid virions would be more efficient for host colonization.

A central question about this mechanism is why BoHV-4 uses alternative splicing of the Bo10 gene instead of expressing gp180 from an unspliced message that would be turned on or off. One of the reasons could be that the unspliced Bo10 message has its own function. Thus, as gp180 interacts with GAGs, the potential soluble form of gp180 could coat the infected cell surface and therefore promote the release of progeny virions by preventing their interaction with cell surface GAGs. Interestingly, a truncated isoform of the major glycoprotein is also secreted from infected cells during EBOLA virus infection [39]. This protein is involved in antibody evasion as it not only serves as a decoy for adsorbing preexisting neutralizing antibodies [40], [41] but also contributes to antigenic subversion of the host immune repertoire [41]. As antibody evasion is particularly important for gammaherpesviruses, the potential soluble form of BoHV-4 gp180 could share similar roles. This hypothesis would imply that gp180 itself is a neutralization target. At the moment, this is not known. This will therefore deserve future studies.

Similar mechanisms could occur in KSHV, where the gp180 homolog is encoded by K8.1. As observed for BoHV-4 Bo10, K8.1 displays several alternative spliced forms [20]. Two proteins, K8.1A and B, are generated from spliced message and encode transmembrane glycoproteins that bind to cell surface heparan sulfate [24], [42]. These proteins are thought to be involved in the initial steps of virion attachment. K8.1 also encodes a third unspliced message, K8.1γ [20], which might likewise code for a soluble protein. Interestingly, a novel alternative message has also recently been described for the EBV BLLF1 gene, encoding gp350/220 [43]. The potential protein encoded by this product also lacks a transmembrane domain. The usage of alternative splicing could therefore allow EBV and KSHV to regulate incorporation of their respective g180 homologs in a similar way to what we showed for BoHV-4 although other regulation mechanisms may exist. This phenomenon and the potential functional consequences on EBV or KSHV tropism will have to be tested in the future.

Deciphering the protein coding complexity of herpesviruses is far from complete. Numerous reports point to a major role for regulated use of alternative splicing in enabling tight temporal control of protein expression and allowing multiple distinct polypeptides to be generated from a single genomic locus [43]–[47]. However, the roles of these splicing events remain largely unknown. Our results show that alternative splicing of the BoHV-4 Bo10 gene orientates viral tropism and determines virion sensitivity to neutralizing antibodies.

## Materials and Methods

### Viruses

The BoHV-4 V.test strain (WT), was initially isolated from a case of orchitis [48]. Bo10 Del, Bo10 Stop and the corresponding revertant strains have been described previously [22], [30]. The other viruses were derived from a cloned BoHV-4 BAC (WT BAC) [49]. Unless stated otherwise, the viruses were grown on MDBK cells.

### Cells

MDBK (ATCC CCL-22) and BoMac [50] cells were cultured in Dulbecco's modified Eagle Medium (Invitrogen) containing 10% fetal calf serum (FCS), Penicillin (200 U/mL)/Streptomycin (200 µg/mL) (Invitrogen) and non-essential amino acids (Invitrogen) diluted following the manufacturer's recommendations. Bovine and rabbit PBMCs were prepared as described elsewhere [22]. Briefly, PBMCs were isolated from 10 ml of blood. Mononuclear cell suspensions were prepared with Ficoll-Paque Premium density gradient media (GE Healthcare) as follows. Cell suspension in sterile PBS was overlaid onto a 7 ml Ficoll-Paque density cushion and centrifuged (1825×*g*) for 20 min at room-temperature. Mononuclear cells at the interface were collected and washed twice in ice-cold PBS before further analysis. PBMCs were cultured in RPMI Glutamax Medium containing 10% FCS, Penicillin (200 U/mL)/Streptomycin (200 µg/mL) (Invitrogen), non-essential amino acids (Invitrogen) diluted following the manufacturer's recommendations, 1 mM Sodium pyruvate, 25 mM HEPES and 50 µM 2-mercaptoethanol.

### Antibodies, sera and reagents

For detection of gp180, encoded by the Bo10 spliced transcript, on western blotting, we used a rabbit monospecific polyserum raised against the C-term end of the gp180 protein (anti-Bo10-c15) [22]. Therefore, this polyserum does not recognize the potential protein encoded by the Bo10 unspliced transcript. The mouse monoclonal antibodies (mAbs) 29 and 35 were raised against gB [30]. The mouse anti-human CD14 Pacific Blue was purchased from Serotec. Rabbit anti-BoHV-4 WT polysera were obtained previously [30]. The PKH26 Red Fluorescent Cell Linker (Sigma) was used for general cell membrane labeling.

### RT-PCR

Sub-confluent monolayers of MDBK cells were infected at a MOI of 1 PFU/cell. 24 hours after infection (p.i.), cytoplasmic RNA was isolated by using RNeasy mini kit (Qiagen). Contaminating DNA was removed by DNase treatment. cDNA was produced by using the First Strand cDNA Synthesis Kit (Roche Applied Science) with poly d(T) primer. The cDNA products were amplified by PCR with *Taq* polymerase (New England Biolabs), and specific primer pairs. Bo10 23-43 (5′-TCATACATTCAAATTGCATGC-3′) and Bo10 839-818 (5′-CATTGAATGAGAACAAACACG-3′) were used to amplify both transcripts. Bo10 PBDF (5′-ATGAGGTTAAGAGTCAGATC-3′) and Bo10 IntronRev (5′-ACCATTTAGTCAAATTCCACAC-3′) were used to amplify unspliced Bo10 product. Bo10 PBDF and Bo10 SplicedOnlyRev (5′-GGATGTCTGTGTGCCTGAG- 3′) were used to amplify the spliced Bo10 product.

### Mutagenesis

We modified the BoHV-4 V.test Bo10 coding sequence (genomic coordinates 65844 to 66743) by BAC mutagenesis [49] to generate two supplemental recombinant viral strains. In the first one (Bo10 MuDir strain), we introduced a single point mutation in the splicing donor site. In the second one, we replaced the entire Bo10 ORF with a sequence devoid of the intron (Bo10 Spliced strain). The Bo10 MuDir and the Bo10 Spliced strains were generated by a two-step mutagenesis procedure in bacteria using the shuttle plasmid pST76KSR [49]. Plasmids to induce homologous recombination were constructed as follows. For the Bo10 MuDir strain, we first PCR-amplified (Platinum Taq DNA Polymerase High Fidelity, Invitrogen) coordinates 65331–67427 of the BoHV-4 V.test genome, including *Sac*I and *Xma*I restriction sites in the respective forward and reverse primers, and T/A cloned the PCR product into the pGEM-T Easy vector (Promega Corporation) resulting in pGEM-T Easy Bo10 zone Rec plasmid. Next, the Bo10 splicing donor site was mutated by site directed mutagenesis (Quikchange site directed mutagenesis, Stratagene) resulting in pGEM-T Easy Bo10 zone Rec MuDir plasmid. For the Bo10 Spliced strain, cDNA was prepared from WT BoHV-4 infected MDBK cells and Bo10 specific sequences were amplified with Bo10 23-43 and Bo10 839-818 specific primers. The lower 740 bp band was then *Bst*BI/*Hpa*I-restricted and cloned into the corresponding sites of the pGEM-T Easy Bo10 zone Rec plasmid resulting in pGEM-T Easy Bo10 zone Rec Spliced plasmid. Each construct was then subcloned as a *Sac*I/*Xma*I fragment into the same sites of the pST76K-SR shuttle vector, and recombined into the BoHV-4 BAC [49]. In the same way, we also isolated revertants in which the Bo10 locus was restored to its wild-type form. Reconstitution of the infectious virus from BAC plasmids was obtained by transfection in MDBK cells.

### Southern blot

Southern blot analysis of viral DNA digested with *Bam*HI was performed with a probe corresponding to nucleotides 65900–66370 of the BoHV-4 V.test strain genome (coding for Bo10 Exon 1) [51].

### Virus purification

BoHV-4 strains grown on MDBK cells were purified as follows. Briefly, after removal of the cell debris by low-speed centrifugation (1,000× *g*, 10 min at 4°C), virions present in the infected cell supernatant were harvested by ultracentrifugation (100,000× *g*, 2 h at 4°C) through a 30% (wt/vol) sucrose cushion. Virions were then banded by isopycnic gradient ultracentrifugation in a 20 to 50% (wt/vol) potassium tartrate gradient in PBS (100,000× *g*, 2 h at 4°C). The band containing virions was collected (∼3 mL), diluted ten fold in PBS and pelleted by ultracentrifugation (100,000× *g*, 2 h at 4°C). The pellet was finally resuspended in PBS, and virus enriched preparations were stored at −80°C.

### Western blot

Purified virions were lysed and denatured by heating (95°C, 5 min) in SDS-PAGE sample buffer (31.25 mM Tris-HCl pH 6.8, 1% (w/v) SDS, 12.5% (w/v) glycerol, 0.005% (w/v) Bromophenol Blue, 2.5% (v/v) 2-mercaptoethanol). Proteins were resolved by electrophoresis on Mini-PROTEAN TGX (Tris-Glycine eXtended) precast 7.5% resolving gels (Bio-Rad) in an SDS-PAGE running buffer (25 mM Tris-base, 192 mM glycine, 0.1% (w/v) SDS) and transferred to polyvinylidene difluoride membranes (Immobilon-P transfer membrane, 0.45 µM pore size, Millipore). The membranes were blocked with 3% non-fat milk in PBS/0.1% Tween-20, and then incubated with the anti-Bo10-c15 rabbit antibodies, with the anti-BoHV-4 polyserum or with the mAb 35 in the same buffer. Bound antibodies were detected with horseradish peroxidase-conjugated goat anti-rabbit IgG pAb or anti-mouse IgG pAb (Dako Corporation), followed by washing in PBS/0.1% Tween-20, development with ECL substrate (GEHealthcare) and exposure to X-ray film.

### Growth curves

The growth kinetics of mutant and revertant viruses were compared to that of WT. Cell cultures were infected at a MOI of 0.01 (multi-step assay). After 1 h of adsorption, the cells were washed and then overlaid with MEM containing 5% FCS. Supernatants of infected cultures and infected cells were harvested together at successive intervals and the amount of infectious virus was determined by plaque assay on MDBK cells [52].

### Immunofluorescence

For PBMCs, staining, washes and incubation steps were performed in FACS buffer (PBS pH 7.4, 0.1% BSA, 0.05% NaN_3_). Cells were incubated with Pacific Blue-conjugated anti-human CD14 (1/50) on ice for 45 min. After washing, cells were analyzed for eGFP and Pacific-Blue fluorescences. Dead cells were excluded with 7-AAD staining. For cell surface staining of viral glycoproteins, cells were infected by the different virus strains at a MOI of 2 PFU/cell for 36 h. After trypsinization and one wash in PBS, the cells were stained with monoclonal antibodies in PBS containing 10% fetal calf serum for 1 h at 4°C. After one wash in PBS, the cells were incubated with goat anti-mouse IgG-Alexa Fluor 633 (Invitrogen) diluted 1∶1,000 in PBS containing 10% FCS for 1 h at 4°C. After one wash in PBS, the cells were analyzed. For intracellular staining, cells were fixed in 1% paraformaldehyde (PFA) for 30 min at room temperature and then permeabilized with 0.1% saponin. Cells were incubated in PBS 10% FCS 0.1% saponin (1 h, 4°C) with the different mAbs specific for BoHV-4 glycoproteins followed by Alexa 633-conjugated goat anti-mouse pAb (Invitrogen). Cells were then washed and analyzed. All the analyses were performed on a FACSAria flow cytometer (Becton Dickinson).

### Neutralization assays

Neutralization was tested by incubating virus with antibody for 1 h at 37°C before adding the virus/antibody mixtures to MDBK cells for a further 3 h. The cells were then overlaid with 0.6% carboxymethylcellulose and the cell monolayers were fixed for plaque counting (based on eGFP expression) after a further 4–5 days. The neutralization assays were carried out with similar amounts of starting virions, in order to ensure that the starting ratio of viral particles to antibodies is similar.

### Quantitative reverse-transcriptase PCR

Total cDNA was produced as described above and analyzed by quantitative PCR with iQ SYBR green supermix (Bio-rad) containing 625 nM of each primer. Quantitative PCR reactions were carried out under the following conditions: initial activation of the *Taq* polymerase (Bio-Rad) at 95°C for 3 min followed by 45 cycles comprising one step of 95°C for 30 sec, one step of 56°C for 45 sec and one step of 72°C for 45 sec. Dissociation curves were performed to check for the presence of a single peak corresponding to the required amplicon.

In parallel, fragments corresponding to both products were quantified. On the one hand, a fragment corresponding to the BoHV-4 Bo10 spliced product, was amplified with the forward primer Bo10 23-43 and the reverse primer Bo10 SplicedOnlyRev. On the other hand, the forward primer Bo10 intron sens (5′GTCCATGTGTGTTAAATCGGG3′) and the reverse primer Bo10 839-818 were used to amplify the specific Bo10 unspliced product. In order to be able to compare amounts of both products, a pGEM-T easy containing specific fragments of both Bo10 transcripts was constructed to establish a unique standard curve. Briefly, we first introduced by T/A-cloning in pGEM-T-easy vector a specific Bo10 spliced sequence generated by PCR on cDNA with Bo10 PBDF and Bo10 Spliced Rev *Bam*HI 5′-**GGATCC**TGGGAGGTTGTGTTGAAGAGT-3′ as primers resulting in pGEM-T spliced vector. Then a sequence specific of Bo10 unspliced product was generated by using the Bo10 MuDir cDNA as a template and Bo10 intron sens *Bam*HI 5′-**GGATCC**GTCCATGTGTGTTAAATCGGG-3′ and Bo10 PBDR *Bam*HI 5′- **GGATCC**TCATAATAAATTATATCCCTGACTATAATT-3′ as primers. This PCR product was restricted with *Bam*HI and ligated into the *Bam*HI site of the pGEM-T spliced vector generating the pGEM-T spliced/unspliced plasmid.

To estimate the proportion of the spliced Bo10 transcript relative to the ORF8 (encoding gB) and ORF47 (encoding gL) transcripts, we amplified the Bo10 spliced as described above and fragments corresponding to BoHV-4 ORF8 and ORF47 with the forward primer 8startfw (5′- CAAATAGTTCATTAGCTGCCTCTCC -3′) and the reverse primer 8middlerev (5′- TCATCAGTAACAGTTGGAATAGTGG -3′), and with the forward primer 47startfw (5′- AAGGATCCGCCGCCACCATGAGAGATATCTATGTTTTTTGT -3′) and the reverse primer 47rev (5′- AACTCGAGCTATAATCTGCCCAGGCCAC -3′) respectively. For these comparisons, we used DNA from the BoHV-4 BAC G Bo10 Spliced plasmid as the unique standard curve.

For each comparison, serial dilutions of the standard curve were made and the same amounts were used in the different reactions. The numbers of copies have been determined based on the measure of the DNA concentration of the standard curve.

### Co-culture experiment

MDBK or BoMac cells in suspension were stained with PKH26 as described by the manufacturer. 1.10^5^ labelled cells were transferred into 6-well plates and then infected with the WT BAC strain at a MOI of 1. Three hours post-infection, cells were washed with acidic solution (PBS pH 3) in order to inactivate and remove the inoculum. 12 hours post infection, 2.10^5^ freshly isolated PBMCs or MDBK controls were added. After 24 hours, the co-cultivated cells were collected with a cell dissociation buffer (Invitrogen) and stained by the Pacific Blue-conjugated anti-CD14 for FACS analysis.

## Supporting Information

Figure S1 **Protein contents of Bo10 MuDir and Bo10 Spliced virions.** Purified virions of the WT BAC, Bo10 MuDir, Bo10 MuDir Rev, Bo10 Spliced and Bo10 Spliced Rev strains were compared for gp180 (with anti-Bo10-c15 serum) and gB (with mAb 35) content per 10^6^ PFU by immunoblotting as described in the Methods. Anti-BoHV-4 polyserum was taken as control. For each blot, the position of a molecular mass (MM) standard (in kDa) is shown on the left.
(TIF)

Figure S2 **Bo10 mRNA splicing determines BoHV-4 entry in CHO GAG− cells.** CHO-K1 cells (CHO GAG+) and the GAG-deficient derivative CHO-pgsA-745 (CHO GAG−) were infected at the MOI of 0.1 with WT BAC (black squares), Bo10 MuDir (open circles), Bo10 MuDir Rev (black circles), Bo10 Spliced (open triangles) or Bo10 Spliced Rev (black triangles) BoHV-4 strains for the times indicated and then washed with PBS. Viral infection was assayed by measuring eGFP expression 18 h later by flow cytometry. In order to compare the different strains, the data are presented as percentages of the maximal values measured for the WT BAC strain.
(TIF)

Figure S3 **Effect of Bo10 mRNA splicing on rabbit PBMCs infection.** Rabbit PBMCs were infected with WT BAC, Bo10 MuDir, Bo10 MuDir Rev, Bo10 Spliced and Bo10 Spliced Rev strains (1 PFU/cell). Twenty-four hours later, cells were analyzed by flow cytometry for CD14 and viral eGFP expression as described in the Methods. The data presented are the average ± SEMs for 6 measurements and were analyzed by 1way ANOVA and Bonferroni posttests, *** p<0.001.
(TIF)

Figure S4 **Relative expression of the spliced Bo10 mRNA in MDBK and BoMac cells.** MDBK and BoMac cells were infected with the BoHV-4 V. test strain at a MOI of 1. Twenty-four hours p.i., relative expressions of Bo10 spliced *vs* ORF47 (gL) transcripts were estimated as described in the Methods. The data presented are the average ± SEMs for 3 measurements and were analyzed by Student's t-test, ** p<0.01.
(TIF)

## References

[1] Henle G, Henle W, Clifford P, Diehl V, Kafuko GW, et al (1969) Antibodies to Epstein-Barr virus in Burkitt's lymphoma and control groups. J Natl Cancer Inst 43: 1147–1157. 5353242

[2] Verma SC, Robertson ES (2003) Molecular biology and pathogenesis of Kaposi sarcoma-associated herpesvirus. FEMS Microbiol Lett 222: 155–163. 12770701 10.1016/S0378-1097(03)00261-1

[3] Young LS, Rickinson AB (2004) Epstein-Barr virus: 40 years on. Nat Rev Cancer 4: 757–768. 15510157 10.1038/nrc1452

[4] Mesri EA, Cesarman E, Boshoff C (2010) Kaposi's sarcoma and its associated herpesvirus. Nat Rev Cancer 10: 707–719. 20865011 10.1038/nrc2888 PMC4721662

[5] Heldwein EE, Krummenacher C (2008) Entry of herpesviruses into mammalian cells. Cell Mol Life Sci 65: 1653–1668. 18351291 10.1007/s00018-008-7570-z PMC11131758

[6] Connolly SA, Jackson JO, Jardetzky TS, Longnecker R (2011) Fusing structure and function: a structural view of the herpesvirus entry machinery. Nat Rev Microbiol 9: 369–381. 21478902 10.1038/nrmicro2548 PMC3242325

[7] Gillet L, May JS, Colaco S, Stevenson PG (2007) Glycoprotein L disruption reveals two functional forms of the murine gammaherpesvirus 68 glycoprotein H. J Virol 81: 280–291. 17050601 10.1128/JVI.01616-06 PMC1797276

[8] Hutt-Fletcher LM (2007) Epstein-Barr virus entry. J Virol 81: 7825–7832. 17459936 10.1128/JVI.00445-07 PMC1951282

[9] Tanner J, Weis J, Fearon D, Whang Y, Kieff E (1987) Epstein-Barr virus gp350/220 binding to the B lymphocyte C3d receptor mediates adsorption, capping, and endocytosis. Cell 50: 203–213. 3036369 10.1016/0092-8674(87)90216-9

[10] Shannon-Lowe CD, Neuhierl B, Baldwin G, Rickinson AB, Delecluse HJ (2006) Resting B cells as a transfer vehicle for Epstein-Barr virus infection of epithelial cells. Proc Natl Acad Sci U S A 103: 7065–7070. 16606841 10.1073/pnas.0510512103 PMC1459019

[11] Turk SM, Jiang R, Chesnokova LS, Hutt-Fletcher LM (2006) Antibodies to gp350/220 enhance the ability of Epstein-Barr virus to infect epithelial cells. J Virol 80: 9628–9633. 16973566 10.1128/JVI.00622-06 PMC1617223

[12] Spriggs MK, Armitage RJ, Comeau MR, Strockbine L, Farrah T, et al (1996) The extracellular domain of the Epstein-Barr virus BZLF2 protein binds the HLA-DR beta chain and inhibits antigen presentation. J Virol 70: 5557–5563. 8764069 10.1128/jvi.70.8.5557-5563.1996 PMC190515

[13] Mullen MM, Haan KM, Longnecker R, Jardetzky TS (2002) Structure of the Epstein-Barr virus gp42 protein bound to the MHC class II receptor HLA-DR1. Mol Cell 9: 375–385. 11864610 10.1016/s1097-2765(02)00465-3

[14] Kirschner AN, Sorem J, Longnecker R, Jardetzky TS (2009) Structure of Epstein-Barr virus glycoprotein 42 suggests a mechanism for triggering receptor-activated virus entry. Structure 17: 223–233. 19217393 10.1016/j.str.2008.12.010 PMC3085316

[15] Wang X, Kenyon WJ, Li Q, Mullberg J, Hutt-Fletcher LM (1998) Epstein-Barr virus uses different complexes of glycoproteins gH and gL to infect B lymphocytes and epithelial cells. J Virol 72: 5552–5558. 9621012 10.1128/jvi.72.7.5552-5558.1998 PMC110204

[16] Li Q, Spriggs MK, Kovats S, Turk SM, Comeau MR, et al (1997) Epstein-Barr virus uses HLA class II as a cofactor for infection of B lymphocytes. J Virol 71: 4657–4662. 9151859 10.1128/jvi.71.6.4657-4662.1997 PMC191687

[17] Wang X, Hutt-Fletcher LM (1998) Epstein-Barr virus lacking glycoprotein gp42 can bind to B cells but is not able to infect. J Virol 72: 158–163. 9420211 10.1128/jvi.72.1.158-163.1998 PMC109360

[18] Borza CM, Hutt-Fletcher LM (2002) Alternate replication in B cells and epithelial cells switches tropism of Epstein-Barr virus. Nat Med 8: 594–599. 12042810 10.1038/nm0602-594

[19] Hutt-Fletcher LM, Chesnokova LS (2010) Integrins as triggers of Epstein-Barr virus fusion and epithelial cell infection. Virulence 1: 395–398. 21178476 10.4161/viru.1.5.12546 PMC3265753

[20] Raab MS, Albrecht JC, Birkmann A, Yaguboglu S, Lang D, et al (1998) The immunogenic glycoprotein gp35-37 of human herpesvirus 8 is encoded by open reading frame K8.1. J Virol 72: 6725–6731. 9658120 10.1128/jvi.72.8.6725-6731.1998 PMC109880

[21] Stewart JP, Janjua NJ, Pepper SD, Bennion G, Mackett M, et al (1996) Identification and characterization of murine gammaherpesvirus 68 gp150: a virion membrane glycoprotein. J Virol 70: 3528–3535. 8648686 10.1093/benz/9780199773787.article.b00034574 PMC190227

[22] Machiels B, Lete C, de Fays K, Mast J, Dewals B, et al (2011) The bovine herpesvirus 4 Bo10 gene encodes a nonessential viral envelope protein that regulates viral tropism through both positive and negative effects. J Virol 85: 1011–1024. 21068242 10.1128/JVI.01092-10 PMC3019988

[23] de Lima BD, May JS, Stevenson PG (2004) Murine gammaherpesvirus 68 lacking gp150 shows defective virion release but establishes normal latency in vivo. J Virol 78: 5103–5112. 15113892 10.1128/JVI.78.10.5103-5112.2004 PMC400354

[24] Wang FZ, Akula SM, Pramod NP, Zeng L, Chandran B (2001) Human herpesvirus 8 envelope glycoprotein K8.1A interaction with the target cells involves heparan sulfate. J Virol 75: 7517–7527. 11462024 10.1128/JVI.75.16.7517-7527.2001 PMC114987

[25] Gillet L, Adler H, Stevenson PG (2007) Glycosaminoglycan interactions in murine gammaherpesvirus-68 infection. PLoS One 2: e347. 17406671 10.1371/journal.pone.0000347 PMC1829177

[26] Blasig C, Zietz C, Haar B, Neipel F, Esser S, et al (1997) Monocytes in Kaposi's sarcoma lesions are productively infected by human herpesvirus 8. J Virol 71: 7963–7968. 9311888 10.1128/jvi.71.10.7963-7968.1997 PMC192155

[27] Fabian K, Ivanics R, Terenyi M, Egyed L (2005) Detection of bovine herpesvirus 4 in CD11b+ leukocytes of experimentally infected rabbits. Acta Vet Hung 53: 265–273. 15959985 10.1556/AVet.53.2005.2.12

[28] Zimmermann W, Broll H, Ehlers B, Buhk HJ, Rosenthal A, et al (2001) Genome sequence of bovine herpesvirus 4, a bovine Rhadinovirus, and identification of an origin of DNA replication. J Virol 75: 1186–1194. 11152491 10.1128/JVI.75.3.1186-1194.2001 PMC114024

[29] Thorley-Lawson DA, Poodry CA (1982) Identification and isolation of the main component (gp350-gp220) of Epstein-Barr virus responsible for generating neutralizing antibodies in vivo. J Virol 43: 730–736. 6287039 10.1128/jvi.43.2.730-736.1982 PMC256176

[30] Machiels B, Lete C, Guillaume A, Mast J, Stevenson PG, et al (2011) Antibody evasion by a gammaherpesvirus O-glycan shield. PLoS Pathog 7: e1002387. 22114560 10.1371/journal.ppat.1002387 PMC3219721

[31] Kolset SO (1987) Proteoglycans in normal and neoplastic monocytes. Exp Cell Res 168: 318–324. 3542538 10.1016/0014-4827(87)90004-8

[32] Milho R, Frederico B, Efstathiou S, Stevenson PG (2012) A heparan-dependent herpesvirus targets the olfactory neuroepithelium for host entry. PLoS Pathog 8: e1002986. 23133384 10.1371/journal.ppat.1002986 PMC3486907

[33] Francois S, Vidick S, Sarlet M, Desmecht D, Drion P, et al (2013) Illumination of Murine Gammaherpesvirus-68 Cycle Reveals a Sexual Transmission Route from Females to Males in Laboratory Mice. PLoS Pathog 9: e1003292. 23593002 10.1371/journal.ppat.1003292 PMC3616973

[34] Hadinoto V, Shapiro M, Sun CC, Thorley-Lawson DA (2009) The dynamics of EBV shedding implicate a central role for epithelial cells in amplifying viral output. PLoS Pathog 5: e1000496. 19578433 10.1371/journal.ppat.1000496 PMC2698984

[35] May JS, Coleman HM, Smillie B, Efstathiou S, Stevenson PG (2004) Forced lytic replication impairs host colonization by a latency-deficient mutant of murine gammaherpesvirus-68. J Gen Virol 85: 137–146. 14718628 10.1099/vir.0.19599-0

[36] Frederico B, Milho R, May JS, Gillet L, Stevenson PG (2012) Myeloid infection links epithelial and B cell tropisms of Murid Herpesvirus-4. PLoS Pathog 8: e1002935. 23028329 10.1371/journal.ppat.1002935 PMC3447751

[37] Das SR, Hensley SE, Ince WL, Brooke CB, Subba A, et al (2013) Defining influenza a virus hemagglutinin antigenic drift by sequential monoclonal antibody selection. Cell Host Microbe 13: 314–323. 23498956 10.1016/j.chom.2013.02.008 PMC3747226

[38] Sattentau Q (2008) Avoiding the void: cell-to-cell spread of human viruses. Nat Rev Microbiol 6: 815–826. 18923409 10.1038/nrmicro1972

[39] Sanchez A, Trappier SG, Mahy BW, Peters CJ, Nichol ST (1996) The virion glycoproteins of Ebola viruses are encoded in two reading frames and are expressed through transcriptional editing. Proc Natl Acad Sci U S A 93: 3602–3607. 8622982 10.1073/pnas.93.8.3602 PMC39657

[40] Ito H, Watanabe S, Takada A, Kawaoka Y (2001) Ebola virus glycoprotein: proteolytic processing, acylation, cell tropism, and detection of neutralizing antibodies. J Virol 75: 1576–1580. 11152533 10.1128/JVI.75.3.1576-1580.2001 PMC114066

[41] Mohan GS, Li W, Ye L, Compans RW, Yang C (2012) Antigenic subversion: a novel mechanism of host immune evasion by Ebola virus. PLoS Pathog 8: e1003065. 23271969 10.1371/journal.ppat.1003065 PMC3521666

[42] Birkmann A, Mahr K, Ensser A, Yaguboglu S, Titgemeyer F, et al (2001) Cell surface heparan sulfate is a receptor for human herpesvirus 8 and interacts with envelope glycoprotein K8.1. J Virol 75: 11583–11593. 11689640 10.1128/JVI.75.23.11583-11593.2001 PMC114745

[43] Concha M, Wang X, Cao S, Baddoo M, Fewell C, et al (2012) Identification of new viral genes and transcript isoforms during Epstein-Barr virus reactivation using RNA-Seq. J Virol 86: 1458–1467. 22090128 10.1128/JVI.06537-11 PMC3264377

[44] Stern-Ginossar N, Weisburd B, Michalski A, Le VT, Hein MY, et al (2012) Decoding human cytomegalovirus. Science 338: 1088–1093. 23180859 10.1126/science.1227919 PMC3817102

[45] Gatherer D, Seirafian S, Cunningham C, Holton M, Dargan DJ, et al (2011) High-resolution human cytomegalovirus transcriptome. Proc Natl Acad Sci U S A 108: 19755–19760. 22109557 10.1073/pnas.1115861108 PMC3241806

[46] van Beurden SJ, Gatherer D, Kerr K, Galbraith J, Herzyk P, et al (2012) Anguillid herpesvirus 1 transcriptome. J Virol 86: 10150–10161. 22787220 10.1128/JVI.01271-12 PMC3446616

[47] Jarosinski KW, Osterrieder N (2012) Marek's disease virus expresses multiple UL44 (gC) variants through mRNA splicing that are all required for efficient horizontal transmission. J Virol 86: 7896–7906. 22593168 10.1128/JVI.00908-12 PMC3421677

[48] Thiry E, Pastoret PP, Dessy-Doizé C, Hanzen C, Calberg-Bacq CM (1981) Herpesvirus in infertile bull's testicle. Vet rec 108: 426. 7257133 10.1136/vr.108.19.426

[49] Gillet L, Daix V, Donofrio G, Wagner M, Koszinowski UH, et al (2005) Development of bovine herpesvirus 4 as an expression vector using bacterial artificial chromosome cloning. J Gen Virol 86: 907–917. 15784885 10.1099/vir.0.80718-0

[50] Donofrio G, van Santen VL (2001) A bovine macrophage cell line supports bovine herpesvirus-4 persistent infection. J Gen Virol 82: 1181–1185. 11297693 10.1099/0022-1317-82-5-1181

[51] Palmeira L, Machiels B, Lete C, Vanderplasschen A, Gillet L (2011) Sequencing of bovine herpesvirus 4 v.test strain reveals important genome features. Virol J 8: 406. 21846388 10.1186/1743-422X-8-406 PMC3178527

[52] Lete C, Machiels B, Stevenson PG, Vanderplasschen A, Gillet L (2012) Bovine herpesvirus type 4 glycoprotein L is nonessential for infectivity but triggers virion endocytosis during entry. J Virol 86: 2653–2664. 22205754 10.1128/JVI.06238-11 PMC3302276

