# Supplementary figures and images for: A Gammaherpesvirus Uses Alternative Splicing to Regulate Its Tropism and Its Sensitivity to Neutralization
Source: PLoS Pathog. 2013 Oct 31;9(10):e1003753. doi: 10.1371/journal.ppat.1003753 (PMC3814654; doi:10.1371/journal.ppat.1003753)

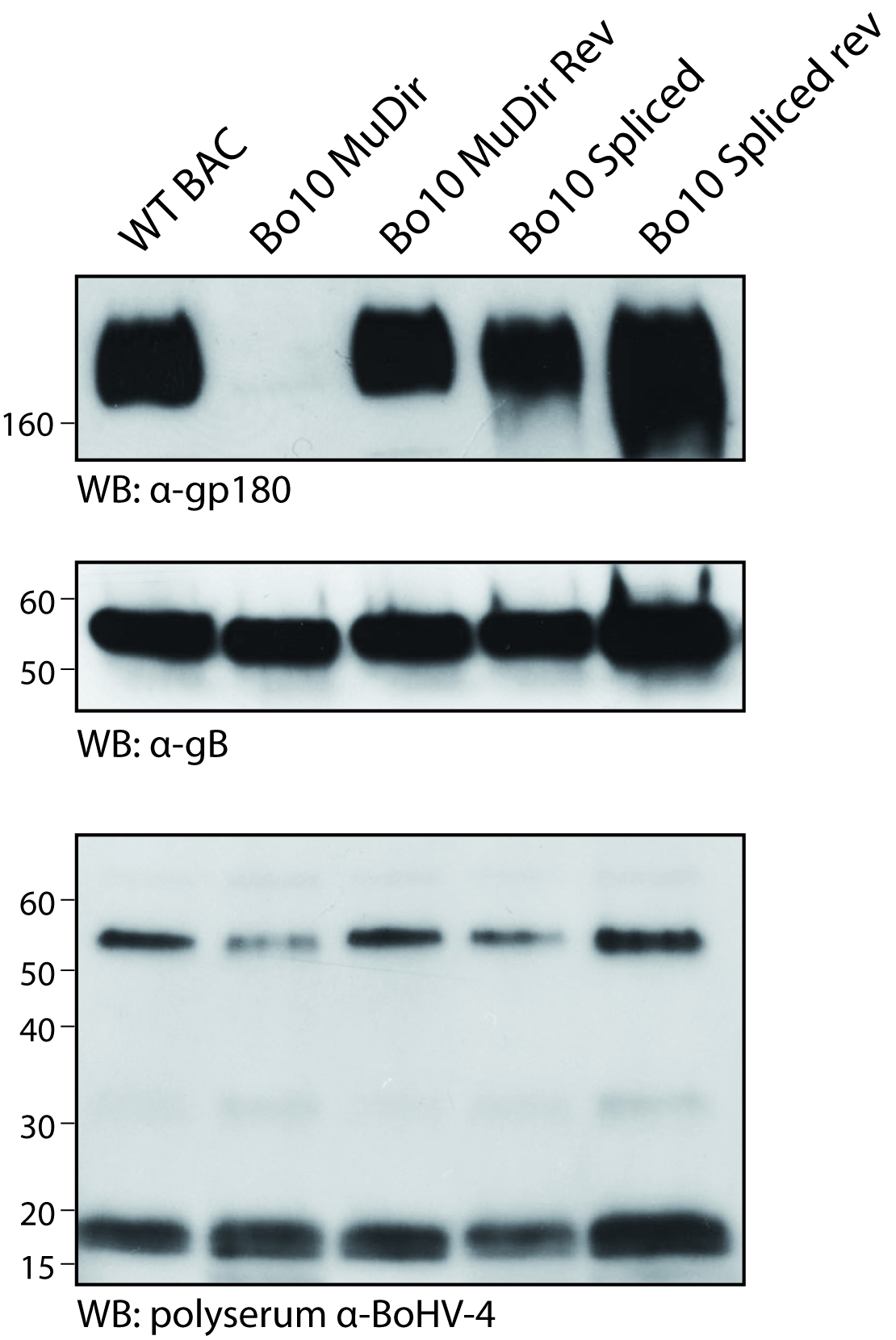

Supplement: Figure S1 — Protein contents of Bo10 MuDir and Bo10 Spliced virions. Purified virions of the WT BAC, Bo10 MuDir, Bo10 MuDir Rev, Bo10 Spliced and Bo10 Spliced Rev strains were compared for gp180 (with anti-Bo10-c15 serum) and gB (with mAb 35) content per 106 PFU by immunoblotting as described in the Methods. Anti-BoHV-4 polyserum was taken as control. For each blot, the position of a molecular mass (MM) standard (in kDa) is shown on the left. (TIF) [file ppat.1003753.s001.tif]

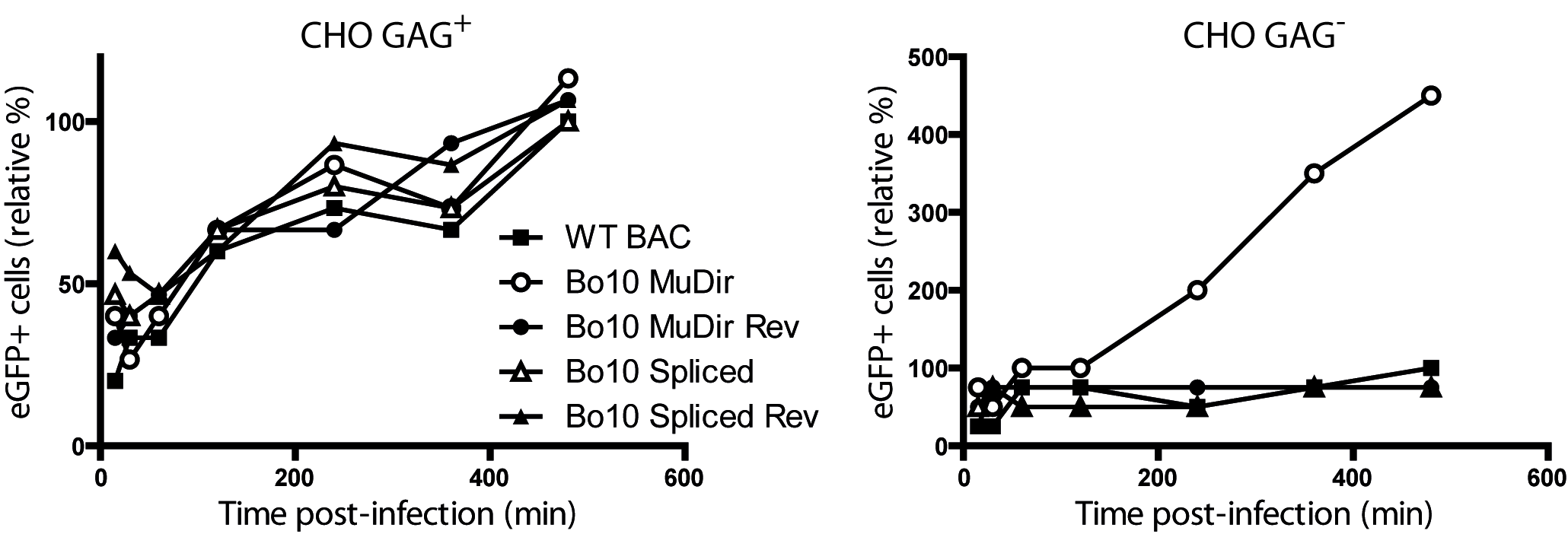

Supplement: Figure S2 — Bo10 mRNA splicing determines BoHV-4 entry in CHO GAG− cells. CHO-K1 cells (CHO GAG+) and the GAG-deficient derivative CHO-pgsA-745 (CHO GAG−) were infected at the MOI of 0.1 with WT BAC (black squares), Bo10 MuDir (open circles), Bo10 MuDir Rev (black circles), Bo10 Spliced (open triangles) or Bo10 Spliced Rev (black triangles) BoHV-4 strains for the times indicated and then washed with PBS. Viral infection was assayed by measuring eGFP expression 18 h later by flow cytometry. In order to compare the different strains, the data are presented as percentages of the maximal values measured for the WT BAC strain. (TIF) [file ppat.1003753.s002.tif]

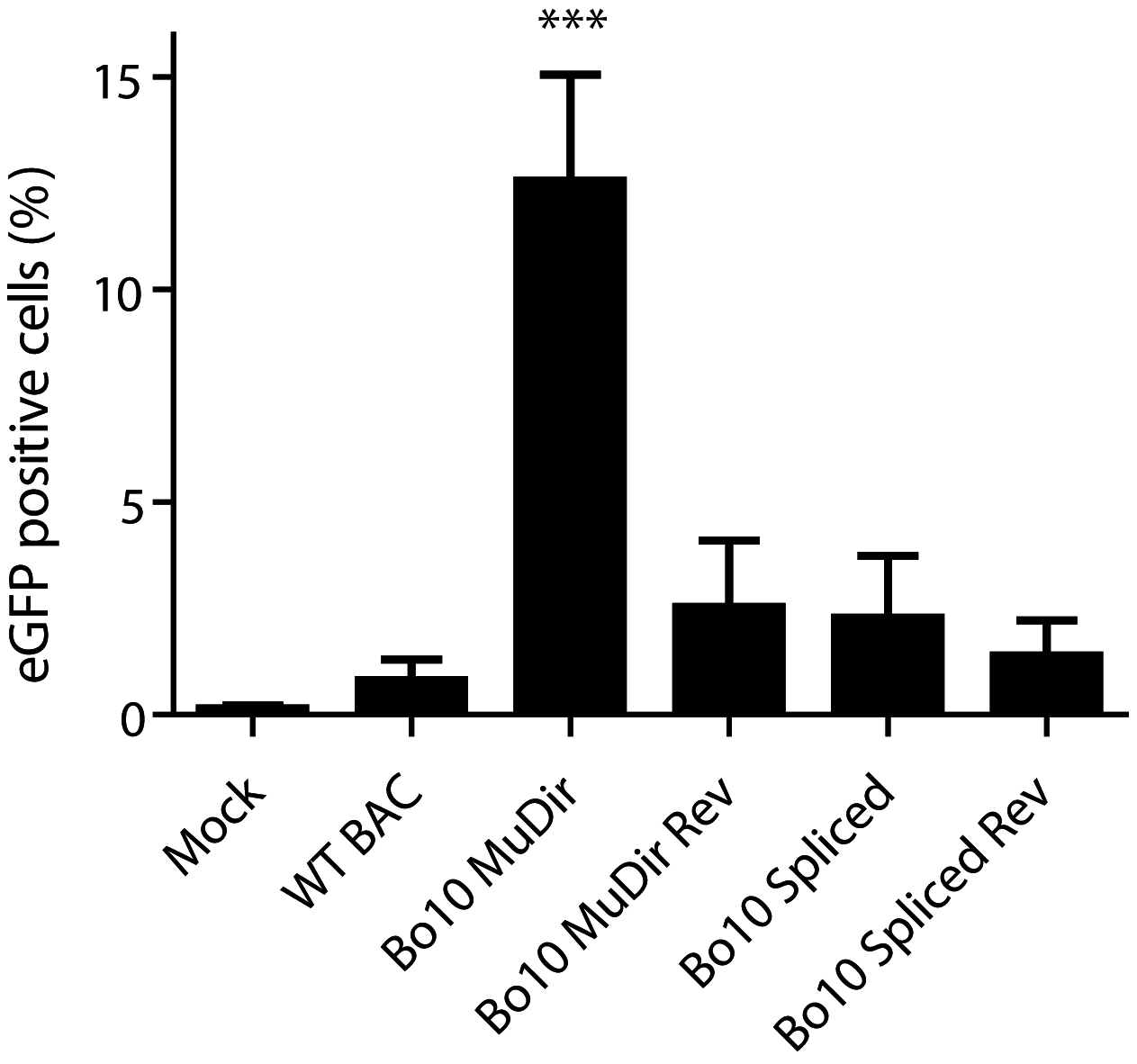

Supplement: Figure S3 — Effect of Bo10 mRNA splicing on rabbit PBMCs infection. Rabbit PBMCs were infected with WT BAC, Bo10 MuDir, Bo10 MuDir Rev, Bo10 Spliced and Bo10 Spliced Rev strains (1 PFU/cell). Twenty-four hours later, cells were analyzed by flow cytometry for CD14 and viral eGFP expression as described in the Methods. The data presented are the average ± SEMs for 6 measurements and were analyzed by 1way ANOVA and Bonferroni posttests, *** p<0.001. (TIF) [file ppat.1003753.s003.tif]

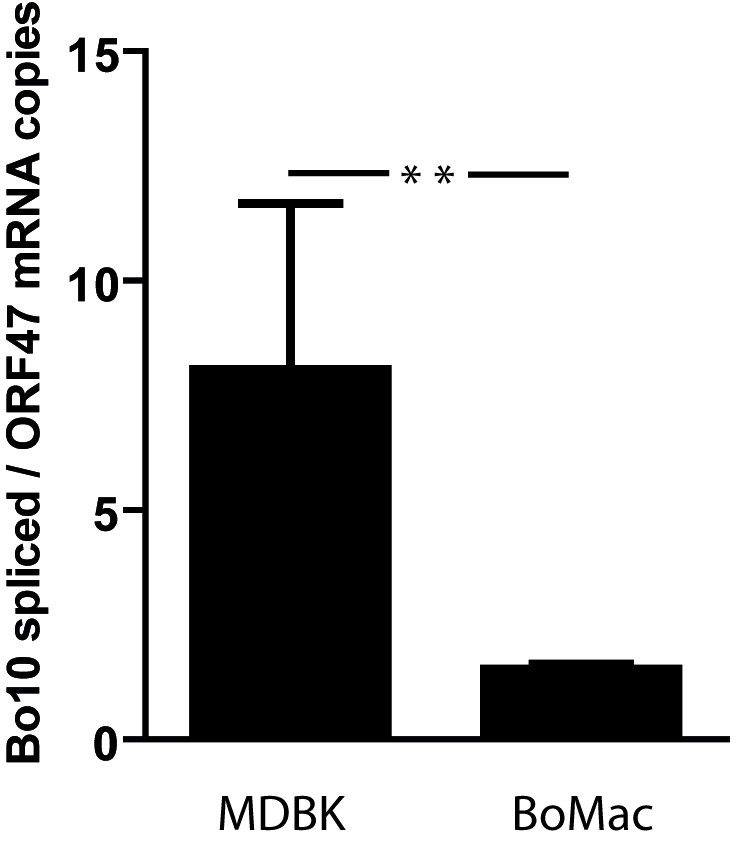

Supplement: Figure S4 — Relative expression of the spliced Bo10 mRNA in MDBK and BoMac cells. MDBK and BoMac cells were infected with the BoHV-4 V. test strain at a MOI of 1. Twenty-four hours p.i., relative expressions of Bo10 spliced vs ORF47 (gL) transcripts were estimated as described in the Methods. The data presented are the average ± SEMs for 3 measurements and were analyzed by Student's t-test, ** p<0.01. (TIF) [file ppat.1003753.s004.tif]
